# Pathogenesis of bronchopulmonary dysplasia: an immunological perspective

**DOI:** 10.3389/fimmu.2026.1965255

**Published:** 2026-10-07

**Authors:** David Riley, Vineet Bhandari

**Affiliations:** 1 Department of Pediatrics, Anne Burnett Marion School of Medicine at Texas Christian University, Fort Worth, TX, United States; 2 Pediatrix Medical Group of Texas — Fort Worth Neonatology, Fort Worth, TX, United States; 3 Division of Neonatology, The Children’s Regional Hospital at Cooper, Cooper Medical School of Rowan University, Camden, NJ, United States

**Keywords:** bronchopulmonary dysplasia, hyperoxia, innate immunity, lung development, macrophage polarization, NLRP3 inflammasome, preterm infant, TLR4

## Abstract

Bronchopulmonary dysplasia (BPD), the most common chronic lung disease of prematurity, is at its mechanistic core an immune-developmental disease in which inflammatory injury disrupts normal lung maturation. In the United States, BPD remains among the most common morbidities of very preterm birth, affecting a substantial proportion of infants born before 32 weeks’ gestation. These infants are born before alveolarization is complete, into an inflammatory environment that can impair ongoing lung development. This review advances a single thesis: the heterogeneous prenatal and postnatal insults that precede BPD (chorioamnionitis and the fetal inflammatory response syndrome, *Ureaplasma* colonization, hyperoxia, mechanical ventilation, sepsis, and intestinal dysbiosis) converge on a small number of innate sensing pathways, principally TLR2 and TLR4 signaling through NF-κB to the NLRP3 inflammasome — with parallel sterile inputs (mitochondrial reactive oxygen species and additional pattern-recognition receptors) feeding the same inflammasome node. We argue that this convergence is what separates BPD from ordinary neonatal lung injury, and that the outcome depends less on the magnitude of the initiating injurious hits than on failure of the resolution program that should terminate them — a hypothesis supported by convergent preclinical evidence but not yet established in preterm infants. Evidence is assembled across three domains: the cellular substrate (M1-skewed, classically activated–like alveolar macrophages, NETs, Th17/Treg imbalance, deficient transplacental IgG transfer); the mediator network (IL-1β, IL-6, IL-8, TNF-α, and the loss of endogenous brakes — specialized pro-resolving mediators, IL-37, Nrf2, short-chain fatty acids); and the signaling architecture (NF-κB, the NLRP3 inflammasome, and trained immunity). We also discuss the mechanistically supported, druggable nodes and the developmental phase in which each is engageable.

## Highlights

- BPD is best understood as an immune-developmental disease: heterogeneous prenatal and postnatal insults converge on a small number of shared innate signaling circuits, chiefly TLR2/TLR4 → NF-κB → NLRP3.
- We propose that what distinguishes BPD from ordinary neonatal lung injury is less the size of the initiating insult than a failure of inflammatory resolution — the lung’s own anti-inflammatory machinery (specialized pro-resolving mediators, efferocytosis, IL-37, Nrf2) is developmentally immature or actively disabled.
- Developmental timing is itself a biological variable: the same insult produces different consequences depending on the maturational stage of the lung and immune system at which it is received.
- The immune disruption does not end at 36 weeks postmenstrual age; it persists as epigenetic myeloid reprogramming (trained immunity and/or tolerization, depending on the exposure) into childhood and extends beyond the lung to the developing brain.
- Because the dominant pathway, endotype, and developmental stage differ between infants, an agent acting broadly on inflammation is unlikely to be matched to any individual infant’s biology; on mechanistic grounds, effective intervention is therefore likely to require pathway-specific and stage-specific immune modulation.
- This review appraises the mechanistically best-supported, druggable nodes in the cascade across four evidence dimensions and identifies those which have never been measured in the preterm human airway or blood.

## 1 Introduction

BPD is the most common chronic lung disease of prematurity and, six decades after its first description, remains without an approved targeted therapy (1–3). Its incidence has not declined despite advances in neonatal care — antenatal corticosteroids, exogenous surfactant, gentler modes of ventilation, and caffeine — and absolute case numbers may even be rising as intact survival extends to the most immature infants. What has changed is the population in which it occurs: survival now extends to infants delivered at 22 to 24 weeks, at the late canalicular to early saccular transition, when the lung possesses neither alveoli nor a mature capillary bed and the immune system possesses neither a competent adaptive compartment nor a functioning resolution program (2). BPD is therefore not a lung injury that happens to occur in an immature host. It is a collision between an inflammatory response and a developmental program that must run to completion in the same tissue, at the same time, using some of the same signaling molecules.

The organizing claim of this review is that BPD is an immune-developmental disease with a convergent architecture. Convergence, multiple-hit priming, failed resolution, developmental timing, and trained immunity are not independent themes; they are chapters within this single frame, each describing a different facet of the same underlying collision between inflammation and development. The insults that precede it are heterogeneous (chorioamnionitis and the fetal inflammatory response syndrome, *Ureaplasma* colonization, hyperoxia, volutrauma, late-onset sepsis, intestinal dysbiosis), yet they are read by the preterm lung through a small number of innate sensors. Microbial lipoproteins and lipopolysaccharide engage TLR2 and TLR4; hyperoxia, mechanical stretch, and cell death generate endogenous DAMPs — low-molecular-weight hyaluronan and HMGB1 — that engage these same receptors, while other danger signals released by the same injuries (notably mitochondrial DNA) are read through distinct sensors such as TLR9 and the cGAS–STING pathway. Additional sterile inputs (mitochondrial reactive oxygen species (ROS), potassium efflux) converge on the inflammasome itself; both microbial and sterile routes signal, in animal models, through MyD88 and NF-κB to prime and then activate the NLRP3 inflammasome, releasing IL-1β and IL-18 into a tissue whose alveolar septation and vascular growth depend on signals (VEGF, FGF-10, elastin patterning) that these cytokines suppress (2, 4–7). This convergence is what renders an otherwise heterogeneous disease mechanistically tractable: a cascade with a small number of shared nodes can be enumerated, appraised, and, in principle, interrupted. Convergence here denotes a dominant shared route rather than an exclusive one, and it is asserted at the shared MyD88–NF-κB–NLRP3 node, not at the receptor: the sensing layer remains heterogeneous, Ureaplasma lipoproteins engaging TLR2/TLR6 heterodimers and gram-negative LPS engaging TLR4. Later viral exposures are additionally sensed through antiviral pattern-recognition receptors (TLR3, TLR7, and RIG-I–like receptors) signaling via IRF3/7 to type I interferon, an arm parallel to the shared node rather than through it; activation of the inflammasome itself requires a second signal — ATP acting through P2X7, potassium efflux, or mitochondrial stress — beyond NF-κB-dependent priming.

What separates BPD from the self-limited lung inflammation of a mature host, we argue, is not the magnitude of the initiating hit but the fate of the response it provokes. Resolution is an active and biosynthetically demanding program. It requires polyunsaturated fatty acid substrate for specialized pro-resolving mediators (SPMs), efferocytosis-competent macrophages, and a set of endogenous brakes, each examined below, that includes IL-37, Nrf2, the IL-1 receptor antagonist, complement regulators, and microbially derived short-chain fatty acids (SCFAs) (8, 9). Every one of these is deficient, developmentally immature, or actively disabled in the extremely preterm infant. The result is a lung in which inflammation is not merely excessive but unterminated, and in which each round of injury deepens the sensitivity of the tissue to the next. Available evidence supports the hypothesis that it is this failure of resolution, rather than the initial insult, that converts injury into impaired development.

These steps — insult, receptor engagement, shared signaling, failed resolution, and impaired development — recur throughout as the five-step architecture depicted in **Figures 1** and **2**.

**Figure 1:**
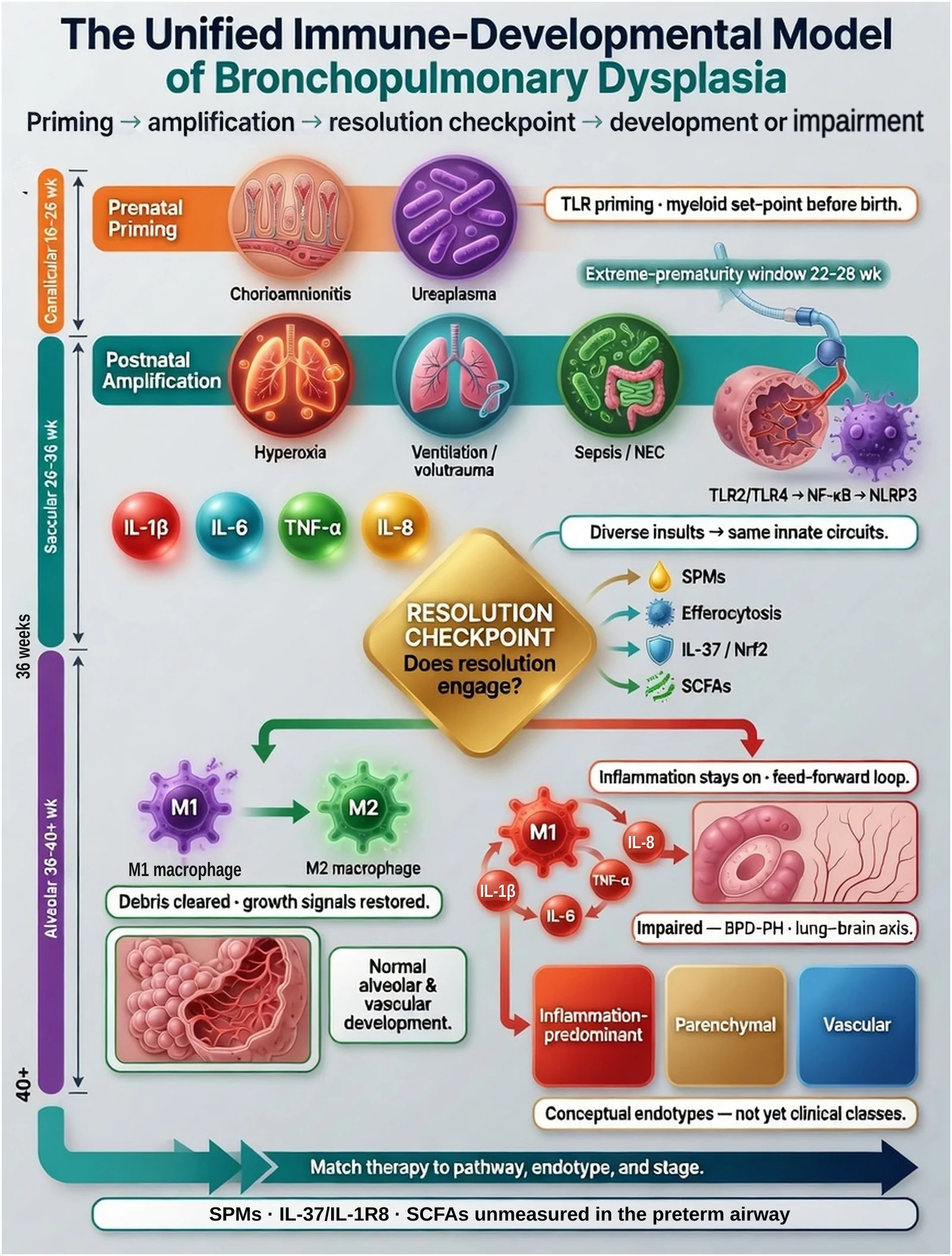
The unified immune-developmental model of bronchopulmonary dysplasia. Prenatal priming (chorioamnionitis, *Ureaplasma*) and postnatal amplification (hyperoxia, mechanical ventilation, sepsis) converge on a principal shared axis — TLR2/TLR4 → NF-κB → NLRP3 signaling, among parallel sterile inputs — in an M1-skewed alveolar macrophage. M1 denotes a dominant activation program rather than a discrete cell type; the depiction is a simplification of a continuum, as stated in Section 5. Whether the resolution checkpoint (specialized pro-resolving mediators, efferocytosis, IL-37/Nrf2, short-chain fatty acids) engages determines the outcome: successful resolution restores normal alveolar and vascular development, while failed resolution produces a self-sustaining inflammatory feed-forward loop and developmental impairment, giving rise to the inflammation-predominant, parenchymal, and vascular endotype pattern — a conceptual framework rather than a validated classification — for which pathway- and stage-matched therapy has been proposed. The vertical band at left marks the developmental stage in which each event occurs — canalicular (16–26 wk), saccular (26–36 wk), and alveolar (36–40+ wk) — with the extreme-prematurity window of 22–28 wk indicated; the bands are sized for legibility and are not drawn to a linear gestational-age scale. TLR, Toll-like receptor; NF-κB, nuclear factor kappa B; NLRP3, NOD-, LRR- and pyrin domain-containing protein 3; SPM, specialized pro-resolving mediator; SCFA, short-chain fatty acid; IL, interleukin; TNF, tumor necrosis factor; IL-1R8 , interleukin-1 receptor 8 (SIGIRR/TIR8); Nrf2, nuclear factor erythroid 2-related factor 2; NEC, necrotizing enterocolitis; BPD-PH, BPD-associated pulmonary hypertension.

**Figure 2:**
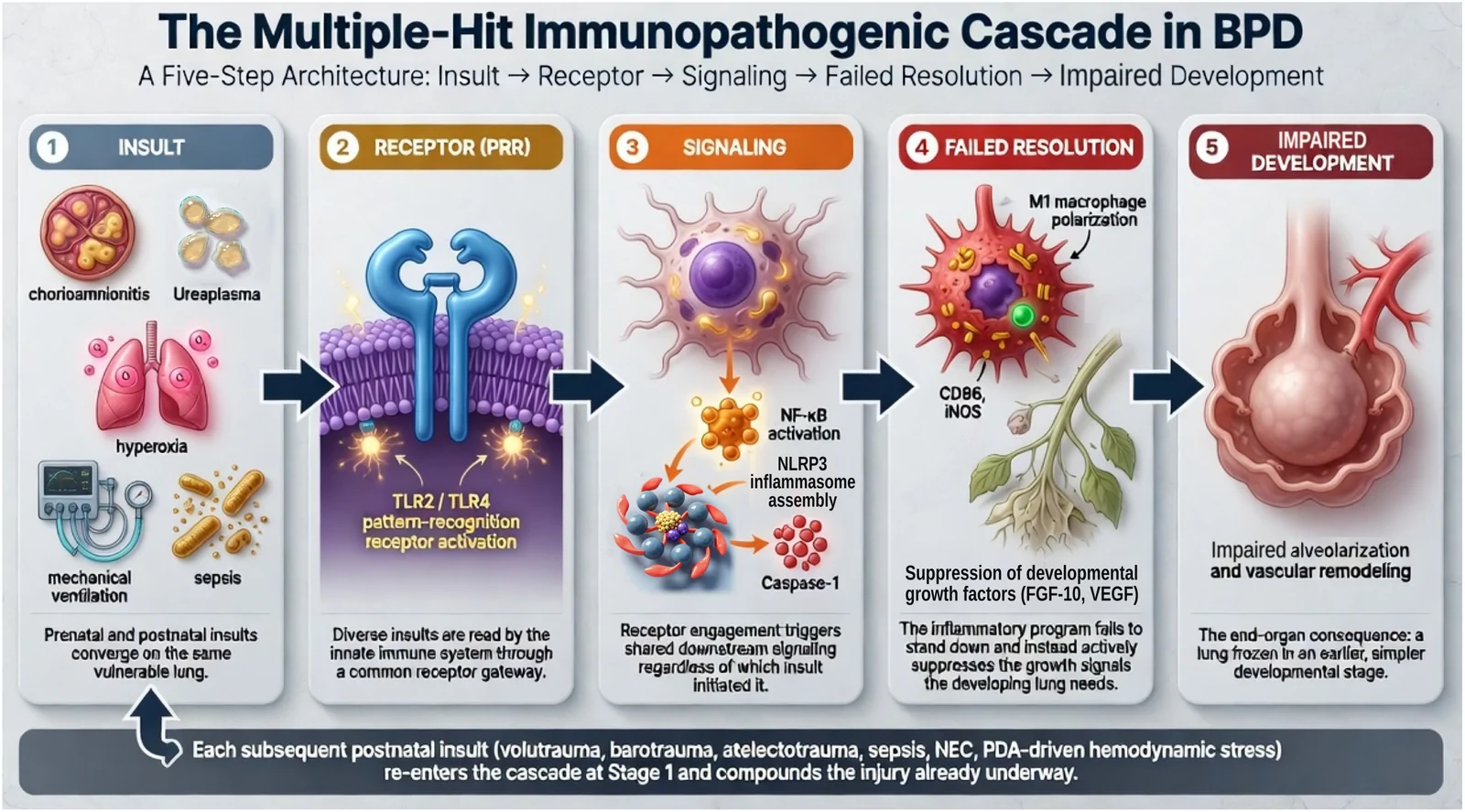
The “multiple hit” immunopathogenic cascade in BPD. Prenatal insults (chorioamnionitis, *Ureaplasma*) and postnatal insults (hyperoxia, mechanical ventilation, sepsis) converge predominantly on TLR2/TLR4 pattern recognition receptor activation, driving NF-κB and NLRP3 inflammasome signaling, M1 macrophage polarization, and suppression of developmental growth factors (FGF-10, VEGF), resulting in impaired alveolarization and vascular remodeling. M1 macrophage polarization denotes a shift in dominant activation program, not conversion between two discrete cell types. TLR, Toll-like receptor; NF-κB, nuclear factor kappa B; NLRP3, NOD-, LRR- and pyrin domain-containing protein 3; FGF, fibroblast growth factor; VEGF, vascular endothelial growth factor; NEC, necrotizing enterocolitis; PDA, patent ductus arteriosus.

## 2 Scope and methods

This article does not follow the conventions of a systematic review. Its purpose is to synthesize current understanding of the core immunopathogenic cascade of BPD — the cellular, mediator, and signaling architecture of the disease — with particular emphasis on moderate-to-severe (broadly Grade 2–3, though the two schemes are not identical) disease. Host factors that modify susceptibility (including sex, receptor polymorphisms, microbiome composition, and endotype) and the long-term, multi-organ trajectory of the immune lesion are introduced here only as they bear on the core cascade; their full development is beyond the scope of this paper. Therapeutic efficacy is deliberately not reviewed here: this paper addresses the immunopathogenic cascade rather than established anti-inflammatory strategies, TLR-directed modulators, mesenchymal stromal cell-derived extracellular vesicles, resolution agonists, or microbiome-directed approaches, together with the questions of timing, endotype enrichment, and trial design on which their evaluation depends. When a mechanism discussed here is targeted by an existing intervention, the intervention is named but not assessed. During finalization, the authors read the cited publications and checked each load-bearing quantitative and mechanistic claim against its primary source; claims the source did not support as stated were corrected or removed. No claim in this review rests on an AI-generated summary of a source. Sources were selected to convey the mechanistic themes most pertinent to the central argument rather than to provide an exhaustive accounting of the field.

Literature searches were conducted using PubMed and the Cochrane Library, supplemented by OpenEvidence (OpenEvidence, Inc., https://www.openevidence.com), an artificial intelligence–powered platform used here as a literature-retrieval aid only. Searches were performed from inception to July 2026 and focused on BPD immunopathogenesis, TLR signaling, macrophage biology, host factors that modify susceptibility, and long-term outcomes in preterm survivors. Selection of sources, the analytical framework, and the synthesis presented here are the authors’ own, and citations refer to the original published literature rather than to platform-generated summaries. Schematic figure artwork was generated from written specifications prepared by the authors, which stipulated the entities, connections, directionality, and labels to be depicted, using Grok 4.5 and 4.6 (xAI, https://grok.com); **Figures 3** and **4** were built directly as vector graphics without an image model, and in the revised figures all label text is typeset programmatically rather than image-generated. The authors checked every figure against its specification and against the manuscript text, correcting or rebuilding those that did not match (see Generative AI statement). The mechanisms synthesized here rest on evidence of very different kinds, so each is assigned in **Table 1** to one or more of four classes: E1, human observational — measured in preterm human airway, blood, or tissue, and associative rather than causal; E2, causal in a neonatal animal model — genetic or pharmacological perturbation with alveolarization or vascular endpoints; E3, *in vitro* mechanistic — cell, explant, or ex vivo systems, including adult human cells; and E4, proposed or extrapolated — inferred from another developmental stage, organ, or disease context. These are categories, not a single ranked ladder: E1 and E2 answer different questions — whether a mechanism operates in preterm humans, and whether it is causal — so the best-supported claims carry both. Claims resting on E3 or E4 alone are identified where they arise. Selection followed stated principles rather than predefined eligibility criteria. Priority went to studies reporting mechanistic data in the developing lung or the preterm human infant, and to studies with alveolarization or vascular endpoints where a causal claim was being made; where a claim rests on adult, non-pulmonary, or non-neonatal data, that provenance is stated in the text and in **Table 1**. Where several studies supported one point, the most directly relevant were preferred over exhaustive enumeration. Appraisal is qualitative, by the classes above and by the four dimensions applied in **Table 2**. Consistent with a narrative rather than systematic review, no protocol was registered, no predefined search string or screening log was kept, no PRISMA flow diagram is reported, and no formal risk-of-bias or GRADE instrument was applied; Section 9.3 states this as a limitation. Abbreviations used most frequently throughout are collected in **Box 1**; a complete alphabetical list appears in the Glossary.

**Figure 3:**
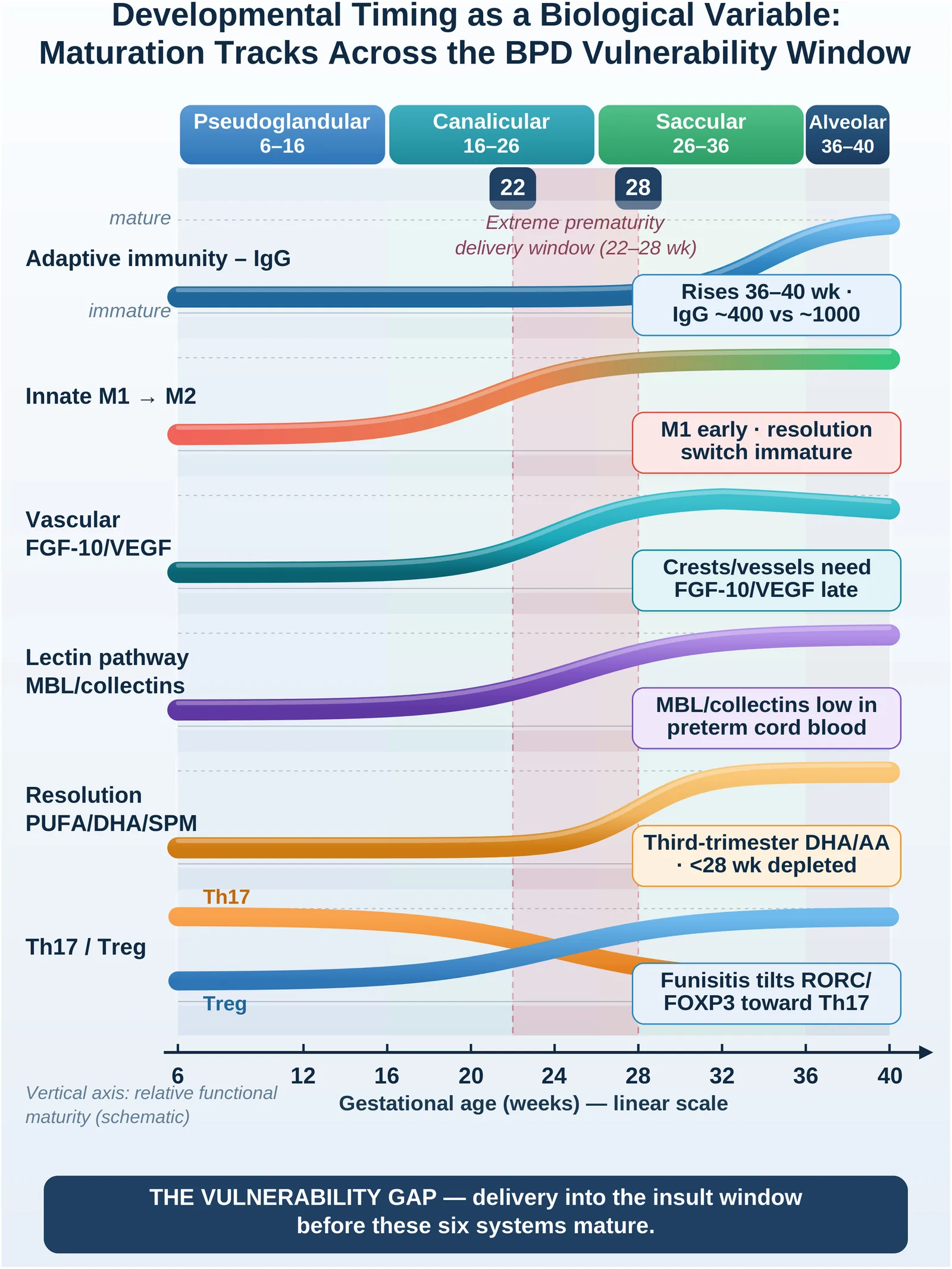
Developmental timing as a biological variable: maturation tracks across the BPD vulnerability window. Six physiological systems relevant to BPD pathogenesis — transplacental IgG transfer, macrophage M1→M2 transition capacity, FGF-10/VEGF-driven vascular growth signaling, lectin-pathway recognition molecules (mannose-binding lectin/collectins), PUFA/DHA substrate for specialized pro-resolving mediator synthesis, and Th17/Treg balance — are plotted across the same gestational-age span (6–40 weeks) as **Figure 5**. At the 22–28 week extreme prematurity delivery window, transplacental IgG transfer and PUFA/DHA substrate accrual remain at or near baseline and the Th17/Treg balance is mid-transition, while the macrophage, vascular and lectin-pathway trajectories, though further advanced, have not reached term maturity; infants are therefore delivered into the insult window with several of their own protective and regulatory systems still incomplete. GA, gestational age; FGF, fibroblast growth factor; VEGF, vascular endothelial growth factor; MBL, mannose-binding lectin; PUFA, polyunsaturated fatty acid; DHA, docosahexaenoic acid; SPM, specialized pro-resolving mediator; Th, T-helper cells; Treg, regulatory T cell; RORC, retinoic acid receptor-related orphan receptor C; FOXP3, forkhead box P3; AA, arachidonic acid. The vertical axis shows relative functional maturity on a schematic scale and does not represent measured values; the gestational-age axis is linear. Evidence class: E1 for transplacental IgG kinetics and preterm cord-blood MBL/collectin concentrations; E3–E4 for the macrophage, vascular, PUFA/DHA and Th17/Treg trajectories, which are schematic syntheses rather than measured series. The macrophage track plots the capacity for M1-to-M2 transition as a continuous developmental variable; M1 and M2 label the ends of that axis and are not two discrete states.

**Figure 4:**
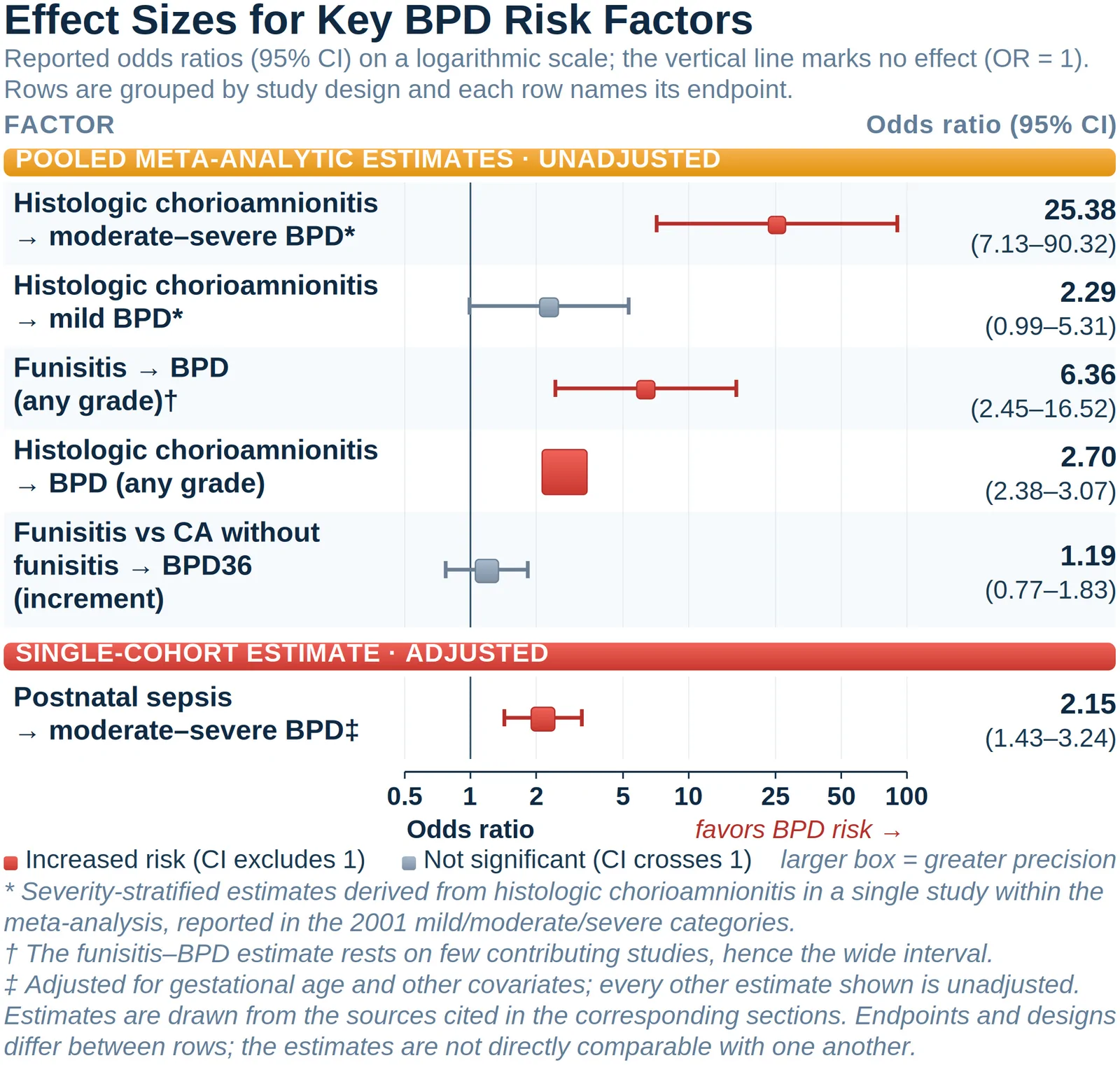
Effect sizes for key BPD risk factors. Reported odds ratios (with 95% confidence intervals) for the principal associations discussed across this review, plotted on a logarithmic scale; the vertical line denotes no effect (OR = 1). Rows are grouped by study design — the pooled, unadjusted meta-analytic estimates first, then the adjusted single-cohort estimate — and each row names the BPD endpoint to which it applies. Because the estimates derive from different designs, different endpoints and different adjustment sets, they are not directly comparable with one another; the figure is intended to show the range and precision of the reported associations rather than to rank them. Antenatal and intrauterine inflammatory exposures (funisitis, histologic chorioamnionitis) carry the largest point estimates; the most extreme severity-stratified estimate (OR 25.38 for moderate-to-severe BPD) derives from histologic chorioamnionitis in a single study rather than from funisitis, reported in the 2001 mild/moderate/severe categories rather than in Jensen grades, and the incremental effect of funisitis over chorioamnionitis alone crosses the line of no effect and remains unadjusted for gestational age. Estimates are drawn from the sources cited in the corresponding sections. BPD36, bronchopulmonary dysplasia at 36 weeks postmenstrual age; OR, odds ratio; CI, confidence interval; CA, chorioamnionitis.

**Table 1:** Mechanistic nodes discussed in Sections 4–9: direction of effect, evidence class, and evidence base.

Mechanistic node (section)	Direction of effect on lung development	Principal evidence base (species/system)	Evidence class	Preterm human airway or blood data?	Refs
Chorioamnionitis/FIRS staging (§4.1)	Fetal inflammatory response increased; positive pooled association with BPD, but the funisitis-specific increment beyond chorioamnionitis is weak once GA is accounted for	Human placental histology; meta-analyses (16 studies/68,397 participants; 158 studies; Bayesian)	E1	Yes — histology and cohort meta-analysis	(15–21)
Ureaplasma vs gram-negative chorioamnionitis (§4.1)	Divergent, not graded: Ureaplasma engages TLR2/TLR6 and TLR9, giving chronic injury with caspase modulation and immune evasion; gram-negative LPS engages TLR4, giving a stronger acute cytokine response	Pulmonary epithelial and endothelial cells *in vitro*; clinical colonization–outcome studies	E1, E3	Partial — colonization associations only; no head-to-head comparison stratified by organism and TLR pathway	(30–33)
FIRS and innate amplification/cross-tolerance (§4.1)	Increased fetal lung TLR2 and TLR4 mRNA, myeloperoxidase and PU.1; repeated exposure instead induces cross-tolerance through IRAK-4 downregulation	Preterm lamb, fetal sheep, rhesus macaque intra-amniotic LPS	E2	No	(22–24)
ACS timing relative to FIRS (§4.1)	Given before LPS, reduces fetal lung inflammation and prevents TLR activation. Given after LPS, does not reverse priming but still improves structural maturation. Combined exposure suppresses ECM development genes	Sheep, rat chorioamnionitis model, rhesus macaque	E2	No	(22, 34–37)
Fetal myeloid epigenetic remodeling (§4.1, §8.2.2)	Net increase in H3K4me3 at intronic and intergenic sites but decreased immune gene expression — tolerization rather than classical training	Human neonatal monocyte ChIP-seq/RNA-seq; SMaRT cord blood analysis	E1, E3	Yes — cord blood and peripheral monocytes	(25, 44, 139)
Transplacental IgG transfer deficit (§4.2)	Cord IgG falls with decreasing GA and pro-inflammatory agalactosylated (G0F) Fc glycoforms rise; cord IgG at or below 4 g/L is associated with more culture-proven sepsis within the same GA stratum	Human preterm cohorts	E1	Yes	(38–43)
Neutrophils and granule proteases (§5)	Earliest cellular responders; elastase, MMPs and ROS degrade matrix and injure epithelium	Human tracheal aspirates; neonatal animal models	E1, E2	Yes	(2, 44)
Macrophage M1 polarization (§5)	Hyperoxia drives M1-like polarization with increased IL-6, TNF-α and GM-CSF; M1 infiltration is a histological feature of the BPD lung; CCR1+ monocytes are upregulated and produce S100A8 and MMP8 via NF-κB and AKT	Neonatal mouse hyperoxia transcriptomics; human BPD lung histology; human airway monocytes	E1, E2	Yes — airway CD14+ and CCR1+ monocytes	(44–49)
Neutrophil extracellular traps (§5.1.1)	Increased in BPD tracheal aspirates; NETs suppress WNT3a and β-catenin, lowering c-MYC, cyclin D and VEGF; histones alone impair alveolarization	Neonatal mouse and rat; human tracheal aspirates	E1, E2	Yes — but day 1–3 levels do not predict BPD, so timing of measurement matters	(44, 50–53)
ILC2s and the IL-33/ST2 axis (§5.1.2)	ILC2-derived IL-13 promotes epithelial-mesenchymal transition in AEC II and AREG acting through EGFR disrupts AEC II transdifferentiation, both giving alveolar simplification; ILC2s also carry reparative potential	Neonatal mouse hyperoxia; human serum	E1, E2	Partial — serum IL-33, CCL24 and CCL13 in severe BPD; ILC2s not measured in the preterm airway	(54–60)
Dendritic cells (§5.1.3)	Increased more than three-fold with chorioamnionitis, ventilation and BPD; Flt3L expands CD103+ cDC1s and Clec9a drives IL-12p40; neonatal DC immaturity favors Th17/Th2 over Th1 polarization	Neonatal mouse hyperoxia and viral challenge; human tracheal aspirates; neonatal DCs *in vitro*	E1, E2, E3	Yes — FLT3L higher in infants who develop BPD, correlating with IL-12 and IFN-γ	(54, 61–66)
NK cells (§5.1.4)	Reduced frequency and cytotoxicity at birth; paradoxical increase in cytotoxic CD56dimCD16+ cells in grade 2–3 BPD; hyperoxia produces a durable shift toward IFN-γ-producing over IL-22-producing NK cells	Human preterm cord blood; neonatal mouse influenza A challenge	E1, E2	Yes — cord blood; MICA and MICB in tracheal aspirates, MICA correlating with FiO_2_	(67–74)
Eosinophils and the type 2 axis (§5.1.5)	IL-5 and IL-13 drive recruitment and survival; the Th2-eosinophilic signature predicts BPD severity from the first week and persists as the asthma-like survivor phenotype	Human cohorts and a phenotype-wide association study; ILC2 model data	E1, E4	Yes — direct effector versus marker role unresolved	(57, 58, 75, 76)
Th17/Treg imbalance (§6)	In models, Th17 cells and IL-17A rise while Tregs and IL-10 fall. In preterm infants Treg frequencies paradoxically rise before BPD, with a hyperactivated day-7 phenotype	Hyperoxia mouse model; human preterm blood and tracheal aspirates	E1, E2	Yes	(71, 73, 77–81)
IL-1β, IL-6 and IL-8 (§7)	IL-1β detectable from day 1 and the IL-1β:IL-1Ra ratio predictive; IL-6 trans-signaling through STAT3 impairs AEC II survival and elastic fiber assembly; IL-8 drives neutrophil influx	Human tracheal aspirates and serum	E1	Yes	(3, 4, 45, 82)
TNF-α and IL-10 (§7)	TNF-α rises by days 21–28, inducing fibroblast proliferation and collagen production, and its SNPs predict onset and severity; reduced IL-10 is consistently associated with BPD	Human cohorts and genetic association studies	E1	Yes	(3, 77, 82)
NLRP3 inflammasome (§8.1)	Hyperoxia activates NLRP3 and caspase-1, releasing mature IL-1β and IL-18; coupled to TLR4 through the TLR4–CD44–NLRP3 axis	Neonatal mouse hyperoxia, including genetic deletion	E2 (E1 limited)	Limited corroboration	(2, 4, 121, 122)
SPM biosynthesis failure (§7.1)	Four separable failures: PUFA substrate depletion; TLR4-driven nuclear-membrane 5-LOX favoring LTB4 over LXA4; efferocytosis failure removing the MerTK feed-forward signal; and loss of RvD2–GPR18 restraint of NLRP3	Adult human monocyte-derived macrophages, murine peritonitis and atherosclerotic lesions; human preterm lipidomics for substrate only	E1, E3, E4	No — no SPM has been quantified in the preterm airway	(8, 9, 83–95)
IL-37/IL-1R8 axis (§7.2)	Suppresses the same cytokine set that is elevated in BPD and inhibits NLRP3 at both the priming and the assembly step	Transgenic mouse and human cell lines; one neonatal lung-injury model, no alveolarization endpoint	E3, E4	No — never measured in a preterm infant; IL-1R8 unmapped in the developing human lung	(97–117)
MerTK-dependent efferocytosis (§7.1)	SPM signaling through FPR2/ALX and MerTK enhances clearance of apoptotic neutrophils; when efferocytosis fails, the feed-forward MerTK signal that drives cytoplasmic 5-LOX translocation and LXA4 synthesis is lost, deepening SPM deficiency	Adult human monocyte-derived macrophages and murine models; receptor-specific rescue demonstrated in preterm-relevant neonatal models	E2, E3, E4	No — efferocytic capacity has not been measured in preterm infants	(9, 93, 94, 147)
Nrf2–Keap1–ARE (§8.2.1)	Nrf2 deficiency increases mortality and worsens alveolar growth inhibition under hyperoxia; pharmacological activation restores alveolar structure	Nrf2-knockout and Keap1-hypomorph neonatal mice, premature rats; adult mice for CDDO-imidazolide	E2	Limited — pathway under-characterized in the human preterm lung	(122–132)
Trained immunity/tolerization (§8.2.2)	Direction is exposure-dependent: neonatal BCG training yields LPS tolerance rather than enhancement, whereas hyperoxia-primed preterm macrophages show an exaggerated LPS response mediated by Egr2 and Gfi1 downregulation	Human neonatal and preterm monocytes and macrophages *in vitro*; cord blood cohorts	E1, E3	Yes — cord blood monocytes	(25, 133–142)
Gut-lung axis and SCFA signaling (§9.1)	SCFAs restrain NF-κB and NLRP3 through HDAC inhibition and GPR43 signaling; the preterm SCFA deficit is imposed by early antibiotic exposure rather than by prematurity alone, and the restraint is context-dependent, reversing under active TLR co-stimulation	Rodent and *in vitro* inflammasome studies; preterm infant microbiome cohorts	E1, E3, E4	Limited — microbiome and metabolic-function data only; SCFA restraint of the neonatal lung inflammasome not shown in preterm infants	(147–149)

For each mechanism the table states the node, the direction in which it acts on alveolar and vascular development, the species and experimental system that generated the evidence, the evidence class or classes as defined in Section 2 (E1, human observational; E2, causal in a neonatal animal model; E3, *in vitro* mechanistic; E4, proposed or extrapolated), and whether the mechanism has been demonstrated in preterm human airway or blood. **Table 2** addresses a different question for the druggable nodes of Section 9.1 — demonstrated necessity, dual alveolar-vascular protection, translational readiness and engageable phase — and the two tables are intended to be read together. Directions are those reported in the cited work and are qualitative; no effect sizes are implied.

**Table 2:** Multidimensional evidence appraisal of the mechanism-to-target nodes.

Node (cascade order)	Necessity demonstrated in an alveolarization model?	Demonstrated in preterm human airway/blood	Dual alveolar + vascular protection (preclinical)	Readiness/human-measurement gap	Engageable phase
TLR4/TLR2 sensing	Yes — TLR4-null neonatal mice protected from impaired alveolarization; adult oxidant-injury data conflict	Yes — TLR pathway polymorphisms; TLR2/*Ureaplasma* arm	Alveolar structure preserved by TLR4 antagonism in preclinical models; vascular endpoints not established	Listed first as the shared entry point, not as the best-validated effector; receptor also carries repair/host-defense signals	Antenatal priming; acute initiation
NLRP3 inflammasome	Yes — NLRP3-null neonatal mice protected; CD44 required	Limited human corroboration	Yes — caspase-1 inhibition protects alveoli, vasculature, and brain	Strongest demonstrated genetic necessity in models; hub where endogenous brakes converge	Acute initiation through amplification
IL-1β/IL-1R signaling	Not directly; effector through which NLRP3 acts	Yes — IL-1β from day 1; elevated in BPD-PH	Yes at 65% O_2_ (not 85%); vascular protection to day 60	Most directly druggable point; developmental/infection-risk caveats apply	Acute initiation through amplification
SPM/FPR2/MerTK efferocytosis	No — four-point failure documented but necessity not tested	No — mediators never measured in preterm infants	Receptor-specific rescue demonstrated preclinically	Follows most directly from the thesis; one descriptive human study from a much stronger position	Amplification/failed resolution
IL-37/IL-1R8 axis	No — the one neonatal lung model used inflammatory and apoptotic endpoints, not alveolarization	No — never measured in a preterm infant	Not established in neonatal lung	Exact convergence with NLRP3 but no human data and no hyperoxia model with alveolarization endpoints	Initiation through amplification
Nrf2–Keap1–ARE	Nrf2 deficiency worsens hyperoxic impairment (model)	Under-characterized in human preterm lung	Antioxidant/anti-pyroptosis protection in neonatal models	Best understood as an upstream brake on inflammasome signal 2, not an independent axis	Acute initiation (± antenatal)
Macrophage polarization/trained immunity	Not applicable — integrating cellular state rather than a discrete node	Yes — M1/tolerization signatures in human samples	Indirect	Excellent pharmacodynamic readout, difficult primary target; drives durability of the lesion	All phases
Gut-lung/SCFA signaling	No — mechanism (HDAC inhibition, GPR43 restraint of NF-κB/NLRP3) shown outside neonatal lung models	Limited direct human data	Indirect	Uniquely modifiable without a drug (antibiotic/feeding practice); translational value exceeds preclinical strength	From first postnatal days

For each node: whether it has been shown necessary (not merely present) in a neonatal lung-injury model with alveolarization endpoints; whether it has been demonstrated in preterm human airway or blood; whether its perturbation protects vascular as well as alveolar development in preclinical models; its readiness for translation and the principal human-measurement gap; and the developmental phase in which it is engageable. Grades are qualitative and reflect the narrative synthesis of this review rather than a formal (e.g., GRADE) appraisal. The nodes are not ranked: they are listed in the order in which they arise in the cascade, the dimensions are not commensurable, and no summary score is computed.

Box 1 **Key abbreviations. Abbreviations used most frequently in this review, grouped by role in the cascade. A complete alphabetical list of all abbreviations appears at the end of the manuscript.**

***Disease and clinical:*** BPD, bronchopulmonary dysplasia; FIRS, fetal inflammatory response syndrome; FIR/MIR, fetal/maternal inflammatory response; GA, gestational age; PMA, postmenstrual age; ACS, antenatal corticosteroid.

***Sensing and adaptors:*** TLR, Toll-like receptor; PAMP, pathogen-associated molecular pattern; DAMP, damage-associated molecular pattern; LPS, lipopolysaccharide; RIG-I, retinoic acid-inducible gene I; MyD88, myeloid differentiation primary response 88; TRIF, TIR-domain-containing adaptor protein inducing interferon-β.

***Signaling:*** NF-κB, nuclear factor kappa B; NLRP3, NOD-, LRR-, and pyrin domain-containing protein 3 (inflammasome); ASC, apoptosis-associated speck-like protein containing a CARD; Nrf2, nuclear factor erythroid 2-related factor 2; Keap1, Kelch-like ECH-associated protein 1; IL-1R8, interleukin-1 receptor 8 (SIGIRR/TIR8).

***Cells:*** M1/M2, classically/alternatively activated macrophage; NET, neutrophil extracellular trap; ILC2, type 2 innate lymphoid cell; DC, dendritic cell; NK, natural killer cell; Th1/Th2/Th17, T helper 1/2/17 cell; Treg, regulatory T cell; AEC/AEC II, alveolar epithelial cell/type II alveolar epithelial cell.

***Mediators and effectors:*** IL, interleukin (e.g., IL-1β, IL-6, IL-10, IL-37); TNF-α, tumor necrosis factor alpha; IFN, interferon; TGF-β, transforming growth factor beta; VEGF, vascular endothelial growth factor; ROS, reactive oxygen species; SPM, specialized pro-resolving mediator; LXA4, lipoxin A4; LOX, lipoxygenase; SCFA, short-chain fatty acid; PUFA, polyunsaturated fatty acid; DHA/EPA, docosahexaenoic/eicosapentaenoic acid; MerTK, Mer receptor tyrosine kinase; IgG, immunoglobulin G.

## 3 Definition and classification of BPD

BPD is defined by the 2019 evidence-based criteria of Jensen and colleagues, derived from NICHD Neonatal Research Network data, as a requirement for respiratory support at 36 weeks PMA in an infant born before 32 weeks’ gestation, with severity graded by the mode and level of that support rather than by oxygen concentration: Grade 1 (nasal cannula ≤2 L/min), Grade 2 (nasal cannula >2 L/min or non-invasive positive airway pressure), or Grade 3 (invasive mechanical ventilation) (2, 10). This updated classification was proposed as an alternative to the Jobe-Bancalari criteria (2001) (11), which remain in wide use, by replacing the oxygen-concentration–based mild/moderate/severe scheme with mode-of-support Grades 1–3 (Grade 1 still denotes milder disease), thereby reducing subjectivity of the FiO_2_-only grading and anchoring severity to the mode and level of respiratory support. A physiological definition of BPD also exists, in which oxygen dependency at 36 weeks PMA is confirmed by a room-air challenge; its utility in contemporary cohorts is limited, and it was not applied in the NICHD severity analysis (10). BPD defined at 36 weeks PMA represents only a snapshot of a dynamic disease: a meaningful subset of infants classified as Grade 1 at this point continue to progress to more severe disease, while others improve.

The mechanistic scope of this review encompasses the full BPD spectrum as defined above but focuses particularly on moderate-to-severe disease, where immunopathological mechanisms are most clearly operative and where the unmet therapeutic need is greatest. Current management remains largely supportive.

The following sections synthesize current mechanistic evidence, tracing the arc from early innate immune priming through emerging translational strategies, to provide a framework for rational therapeutic development.

## 4 The “multiple hit” model and immune priming

BPD pathogenesis follows a “multiple hit” paradigm (**Figure 2**) — a cascade of inflammatory insults that, taken individually, a mature lung might resolve, but that overwhelm the preterm lung’s rudimentary self-regulatory architecture before its structural and immunological foundations are complete (**Figure 5**). Disease emerges when cumulative inflammatory burden exceeds the resolving capacity available at a given developmental stage — an inflammatory-developmental threshold crossed not by insult magnitude alone but by the ratio of injury to the immaturity of the tissue that receives it. These insults occur during the late canalicular to early saccular window (approximately 22–28 weeks GA) (2). The first hits are prenatal: chorioamnionitis, present in the great majority of placentas delivered at 21–24 weeks’ gestation (≈94%) and in a substantial minority thereafter, against 3–5% at term (12), exposes the developing fetal lung to microbial ligands and inflammatory cytokines weeks before birth, activating FIRS and upregulating innate immune mediators in a system not yet equipped to resolve them. *Ureaplasma* species colonization is particularly implicated; bacterial lipoproteins are sensed through TLR2, and this prenatal signaling primes the neonatal immune landscape before the first breath is drawn (2). The receptor-level evidence is presented in Section 4, and macrolide prophylaxis has been trialed in ventilated preterm neonates, in whom its rationale is anti-inflammatory as well as antimicrobial (13). Critically, these hits are not simply additive: each exposure reprograms the immune response to the next, so that the sequence and timing of insults, not their number alone, shape the resulting phenotype.

**Figure 5:**
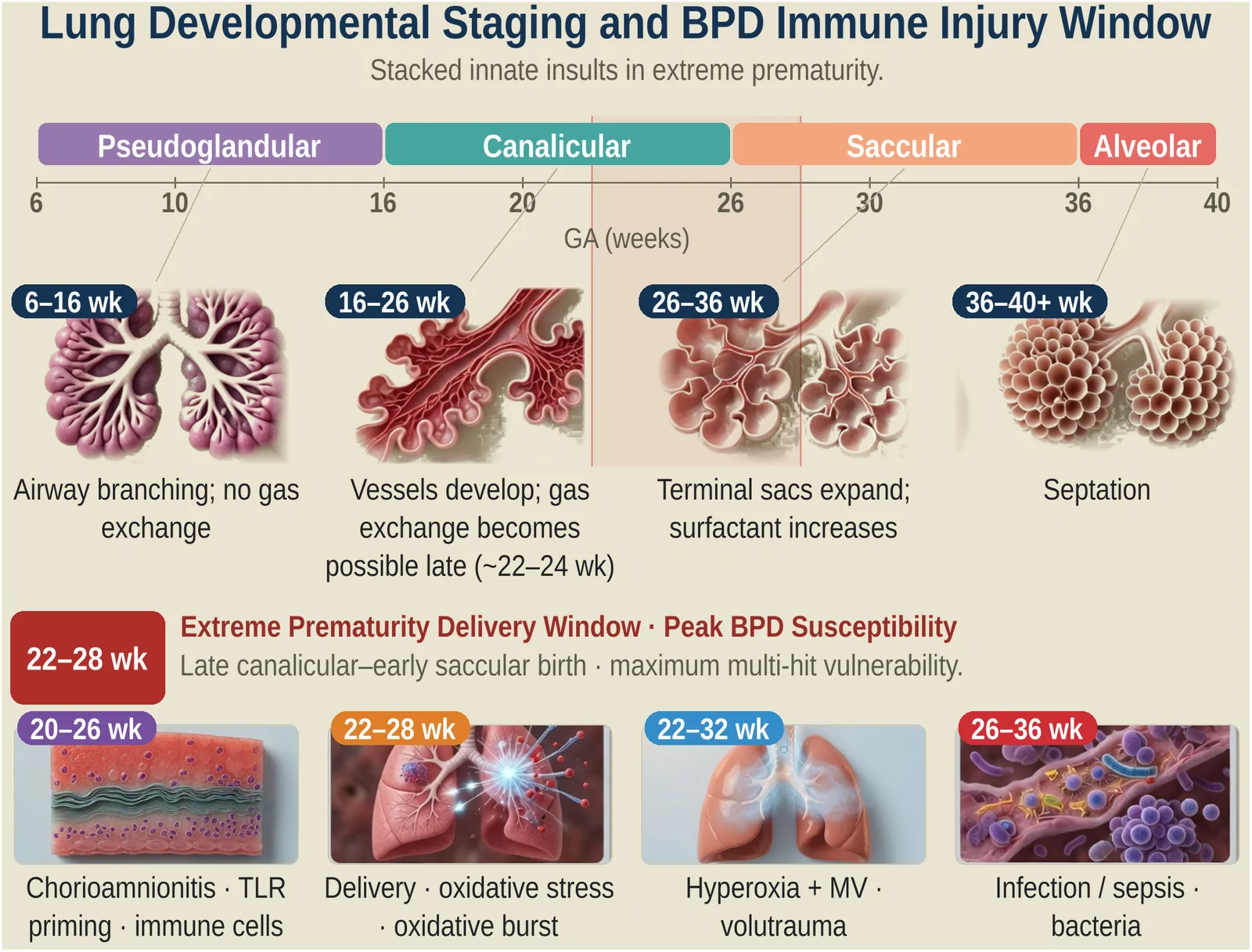
Lung developmental staging and BPD immune injury window. Horizontal timeline depicting the four canonical stages of human lung development from 6 to 40+ weeks gestational age (GA). The extreme prematurity delivery window (22–28 weeks GA) coincides with the late canalicular to early saccular transition—the period of maximum vulnerability to immune-mediated alveolar injury. Annotations indicate the timing of the major “multiple hit” insults: chorioamnionitis (prenatal TLR2/TLR4 priming), delivery into the late canalicular–early saccular transition with associated oxidative stress, postnatal hyperoxia and mechanical ventilation, and nosocomial infection. Extreme prematurity (22–28 weeks GA) spans late canalicular through early saccular development. BPD, bronchopulmonary dysplasia; GA, gestational age; MV, mechanical ventilation; TLR, Toll-like receptor.

Each subsequent postnatal insult compounds the injury already underway. The mechanical forces of ventilation (volutrauma, barotrauma, atelectotrauma) further injure an airway epithelium rendered fragile by prenatal inflammation; hyperoxia generates a sustained oxidant burden that drives sterile TLR4 activation; and postnatal sepsis, necrotizing enterocolitis, and the hemodynamic stress of a patent ductus arteriosus each add to the cumulative inflammatory load (2, 3) (**Figure 5**).

As **Figures 3** and **5** illustrate, GA at exposure shapes the outcome as much as the identity of the exposure itself.

The inflammation that results is exaggerated and self-perpetuating, transforming the lung microenvironment from a site of active alveolar and vascular development into one of chronic injury — redirecting the biological program from septation and angiogenesis toward alveolar simplification and vascular remodeling (2).

### 4.1 Chorioamnionitis classification, FIRS staging, and downstream immune programming

The designation of chorioamnionitis as the critical “first hit” in BPD pathogenesis obscures an important distinction: not all intrauterine inflammation carries equivalent risk, and the maternal and fetal inflammatory responses represent mechanistically and prognostically distinct entities. The Redline histological grading system provides the precision the clinical literature has lacked by classifying intrauterine inflammation along two independent axes — the maternal inflammatory response (MIR) and the fetal inflammatory response (FIR) — each with three anatomically defined stages (12, 14). The MIR stages progress from acute subchorionitis (Stage 1: neutrophils in subchorionic fibrin), to chorioamnionitis with PMN extension into the fibrous chorion and amnion (Stage 2), to necrotizing chorioamnionitis with karyorrhexis, amniocyte necrosis, and amnion basement membrane changes (Stage 3). The FIR stages progress from chorionic vasculitis or umbilical phlebitis (Stage 1: inflammation limited to the umbilical vein wall), to involvement of the umbilical vein plus one or more umbilical arteries (Stage 2), to necrotizing funisitis with extension of inflammation into Wharton’s jelly and the characteristic concentric “ring” pattern (Stage 3). Each stage is further graded as Grade 1 (not severe) or Grade 2 (severe: near-confluent intramural PMNs with attenuation of vascular smooth muscle) (12).

The distinction matters because the histologic fetal inflammatory response (FIR Stages 1–3) — which begins with chorionic vasculitis or umbilical phlebitis at Stage 1 and progresses to funisitis at higher stages — is the tissue counterpart of the systemic fetal inflammation most directly relevant to BPD immunopathogenesis (15). FIRS itself is defined biochemically by elevated fetal plasma IL-6 (>11 pg/mL) (16) — a threshold established by cordocentesis in preterm gestations, whereas the cord-blood series cited below defined the fetal response by umbilical cord plasma CRP ≥200 ng/mL — and is frequently, but not invariably, accompanied by funisitis and is associated with elevated IL-8 and CRP; serum IL-6 thresholds of ~20 pg/mL at delivery and ~572 pg/mL at 1–3 hours of life discriminate funisitis-positive from funisitis-negative neonates with meaningful sensitivity, and the frequency of FIRS, funisitis, and early-onset neonatal sepsis increases in a stepwise fashion with advancing stage of umbilical cord inflammation (17, 18). A maternal-only Stage 1 or Stage 2 MIR without concurrent FIR reflects neutrophil migration driven by maternal chemokines and does not produce the fetal systemic cytokine surge associated with BPD risk (15, 19) (**Figure 6**).

**Figure 6:**
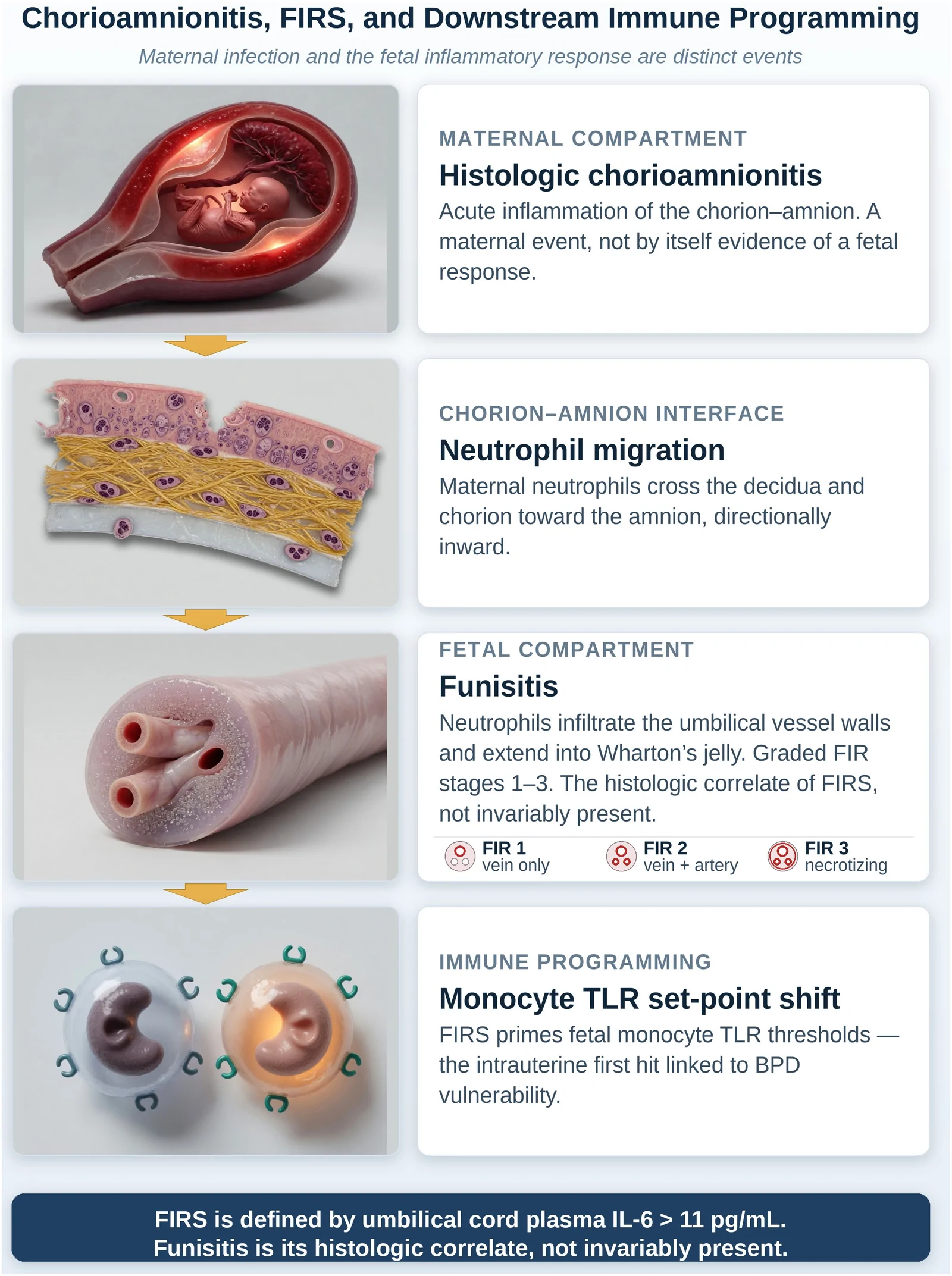
Chorioamnionitis, FIRS staging, and downstream immune programming. Maternal histologic chorioamnionitis is distinguished from the fetal inflammatory response (funisitis, FIR stages 1–3). The three cord cross-sections in the fetal-compartment panel key the Redline FIR stages: stage 1, inflammation limited to the umbilical vein wall; stage 2, the vein plus one or more umbilical arteries; stage 3, necrotizing funisitis, with the concentric perivascular ring in Wharton’s jelly. Fetal inflammatory response syndrome (FIRS) is defined biochemically by elevated fetal plasma IL-6 (>11 pg/mL); funisitis is its histologic correlate but is not invariably present. FIRS is the intrauterine first hit most directly linked to neonatal innate priming (TLR/monocyte set-points) and subsequent BPD vulnerability. FIRS, fetal inflammatory response syndrome; FIR, fetal inflammatory response; IL-6, interleukin-6; TLR, Toll-like receptor.

The quantitative evidence linking funisitis specifically to BPD is suggestive but must be read with care. A 2024 meta-analysis of 16 studies encompassing 68,397 participants reported a positive association between the fetal inflammatory response (funisitis/fetal vasculitis) and BPD (OR 6.36, 95% CI 2.45–16.52), alongside a pooled association for histologic chorioamnionitis and BPD (OR 2.70, 95% CI 2.38–3.07); the broader chorioamnionitis-or-fetal-vasculitis association with lung injury overall was more modest (OR 1.43, 95% CI 1.06–1.92). The very wide confidence interval on the funisitis estimate reflects the small number of contributing studies, and the extreme severity-stratified signals reported in that meta-analysis — a strong association with moderate-to-severe BPD (OR 25.38, 95% CI 7.13–90.32) and a non-significant one with mild BPD (OR 2.29, 95% CI 0.99–5.31) — derive from histologic chorioamnionitis in a single study rather than from funisitis, are reported in the 2001 mild/moderate/severe categories rather than in Jensen grades, and should not be read as a funisitis-specific severity gradient (**Figure 4**) (20). However, a larger JAMA Network Open meta-analysis (158 studies) found that the incremental risk of funisitis beyond chorioamnionitis alone did not reach statistical significance for BPD at 36 weeks (BPD36, defined in that meta-analysis as a supplemental oxygen requirement at 36 weeks PMA) (OR 1.19, 95% CI 0.77–1.83), and a Bayesian meta-analysis similarly concluded that evidence for a funisitis-specific effect on BPD was weak, though the effect on intraventricular hemorrhage was robust (21, 22). This discrepancy is most plausibly explained by confounding through GA — funisitis is more prevalent at earlier gestations, which independently drives BPD severity, making GA adjustment essential for isolating the immunological contribution of the fetal response.

At the mechanistic level, FIRS and funisitis produce downstream effects on fetal innate immunity that extend well beyond the immediate cytokine burst. In a preterm lamb model, intra-amniotic LPS significantly upregulated fetal lung TLR2 and TLR4 mRNA expression and increased myeloperoxidase activity and myeloid transcription factor PU.1 expression in BAL cells, establishing innate immune amplification as an early consequence of FIRS-level intrauterine inflammation (23). In fetal sheep, repeated intra-amniotic LPS exposure modulated blood monocyte responsiveness to TLR2, TLR5, and TLR9 ligands with increased IL-6, TNF-α, and ROS production, but also induced cross-tolerance via IRAK-4 downregulation — a trained immunity/tolerization dichotomy directly relevant to the discussion of trained immunity in Section 8.2 (24). In a rhesus macaque model, intra-amniotic LPS exposure within the physiological range of clinical chorioamnionitis rapidly activated a potent pulmonary myeloid response dominated by neutrophils and macrophages with transcriptional profiles consistent with TLR ligand exposure and IL-1β/TNF-α signaling, evident within days of exposure (25).

Epigenetic remodeling of the fetal myeloid compartment is a particularly consequential downstream effect. Chorioamnionitis exposure is associated with global restructuring of the H3K4me3 histone modification landscape in neonatal monocytes, with H3K4me3 deposition shifted toward intronic and intergenic regions at the expense of immune gene promoters — producing paradoxical immune pathway gene suppression that renders chorioamnionitis-exposed monocytes less responsive to secondary inflammatory challenge (26). This is not trained immunity but its functional opposite: a tolerization phenotype that may simultaneously increase susceptibility to postnatal infection while maintaining a chronic low-grade pro-inflammatory baseline (27). Exposure to moderate-to-severe HCA is independently associated with adverse motor and cognitive development at 22 to 26 months’ corrected age (28). Differential gene expression in HCA-exposed cord blood mononuclear leukocytes encompasses key regulatory genes across inflammatory, immune, respiratory, and neurological pathways (29). Adding cellular specificity, infants exposed to funisitis (but not maternal chorioamnionitis alone) showed elevated *RORC* (the Th17 transcription factor) expression and an elevated *RORC/FOXP3* ratio in peripheral leukocytes persisting over 4 weeks after birth, suggesting that funisitis — rather than maternal chorioamnionitis alone — is the exposure that drives the Th17 polarization bias and Treg suppression detailed in Sections 6 and 8 (30). Collectively, these observations indicate that the principal consequence of FIRS is not transient fetal inflammation but durable reprogramming of innate immune responsiveness — in effect, a new inflammatory set-point that lowers the activation threshold for subsequent postnatal injury.

The distinction between *Ureaplasma*-driven and gram-negative (*E. coli*) chorioamnionitis carries mechanistic significance for BPD because these organisms activate entirely different TLR pathways. *Ureaplasma* lipoproteins, including the multiple-banded antigen (MBA), engage TLR2/TLR6 heterodimers and TLR9 (via bacterial DNA), requiring lipid raft formation and heterotypic receptor complexes (31), while *E. coli* LPS, a canonical TLR4 ligand, drives a markedly stronger acute cytokine response in pulmonary epithelial and endothelial cells (IL-8, and in pulmonary microvascular endothelial cells also TNF-α, IL-6 and IL-1β), which *Ureaplasma* species do not elicit (32). *Ureaplasma* does not simply produce a blunted version of gram-negative inflammation; in adult human cell lines it differentially modulates caspase pathways, downregulating caspase mRNA in alveolar epithelial cells (potentially inhibiting apoptotic clearance and enabling persistent colonization) while increasing caspase activity and cell death in pulmonary endothelial cells (32). The result, in preterm animal models, is a chronic, structurally disruptive inflammation driven by immune evasion, a distinction that may explain why *Ureaplasma* colonization has been inconsistently associated with particularly insidious BPD rather than the acute inflammatory presentation of gram-negative sepsis-associated lung injury (33, 34). Formal head-to-head comparison of BPD immunopathological outcomes stratified by colonizing organism and TLR pathway in human cohorts remains an important and unaddressed evidence gap.

The immunological consequences of FIRS are substantially modified by the timing of ACS administration relative to the inflammatory insult. In preterm lambs, betamethasone administered before intra-amniotic LPS exposure suppressed fetal lung inflammation, prevented TLR-pathway activation, and counteracted LPS-induced changes in TGF-β signaling (including CTGF induction and caveolin-1 downregulation) (23, 35). When administered after LPS (that is, once FIRS is established), betamethasone did not counteract established inflammation but still promoted structural maturation, increasing surfactant and improving gas volumes (23). In a rat chorioamnionitis model, antenatal betamethasone improved long-term lung function (15.3% reduction in total lung resistance, 9.5% improved compliance), preserved alveolarization and vascular growth, and reduced right ventricular hypertrophy by 42.4% (36). In rhesus macaques, combined ACS and LPS exposure produced a “mature-like” transcriptomic profile with TP53-mediated suppression of mesenchymal proliferation, but also suppressed extracellular matrix development genes (*COL1A1, COL1A2, COL3A1, FGF9, FGF10*) — raising the important concern that the short-term respiratory advantage of ACS in the setting of FIRS may come at the cost of abnormal ECM maturation and potentially increased chronic lung disease risk (37). Evidence on whether ACS specifically modifies fetal alveolar macrophage M1/M2 polarization under FIRS conditions remains limited; the most relevant published data address decidual rather than pulmonary macrophage responses, and ACS-modified placental macrophage polarization itself varies with the presence of histologic chorioamnionitis (38) (**Figure 7**).

**Figure 7:**
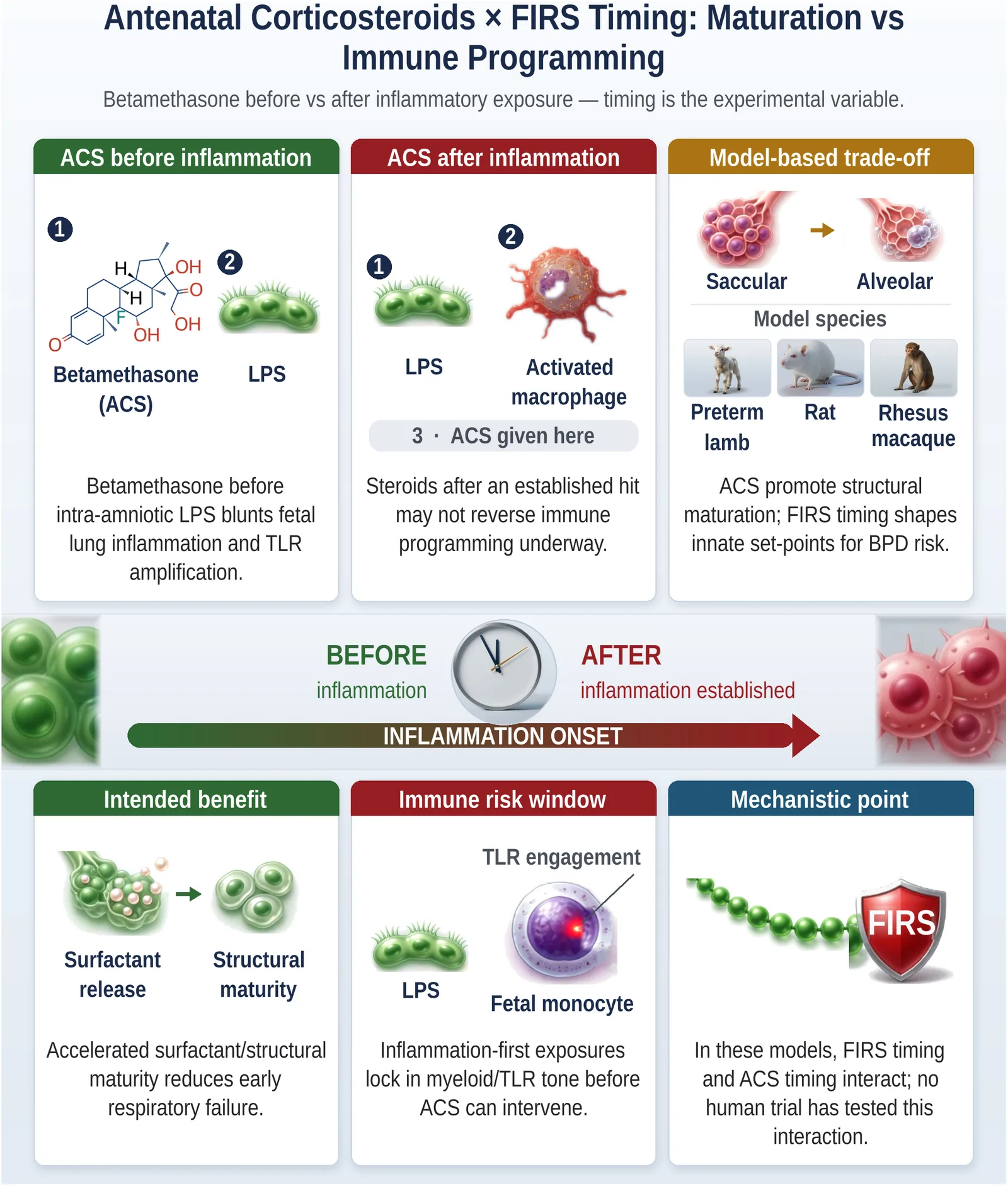
Antenatal corticosteroids × FIRS timing: maturation versus immune programming. In preterm lamb, rat, and rhesus macaque models of intra-amniotic inflammation, betamethasone administered before an inflammatory exposure can suppress fetal lung inflammation and limit TLR-pathway amplification, whereas steroids given after inflammation may not reverse programming already initiated. ACS promote structural maturity, but the timing relative to FIRS shapes innate set-points relevant to later BPD risk. BPD, bronchopulmonary dysplasia; ACS, antenatal corticosteroids; FIRS, fetal inflammatory response syndrome; LPS, lipopolysaccharide; TLR, Toll-like receptor.

### 4.2 Transplacental maternal IgG transfer deficits and immune vulnerability in extremely preterm infants

The immunological vulnerability of extremely preterm infants to pulmonary infection and inflammation (a primary driver of BPD) is substantially amplified by quantitative and qualitative deficits in transplacentally acquired maternal IgG. Transplacental IgG transfer is almost entirely a third-trimester phenomenon: the bulk of maternal-to-fetal IgG transport occurs after 28 weeks’ gestation, with the sharpest acceleration between 36 and 40 weeks (39, 40).

Beyond quantity, IgG quality may be independently relevant. Agalactosylated (G0F) IgG Fc glycoforms, a pro-inflammatory pattern, become more abundant as GA falls (15.5% below 28 weeks vs. 10.4% at term) and are further enriched in infants who develop BPD (16.9% vs. 10.6%, significant in the ≥28-week stratum), suggesting that the inflammatory quality of transplacentally acquired IgG, not only its quantity, may modulate pulmonary inflammatory risk (41).

The clinical corollary is direct: within the same GA stratum (26–29 weeks), infants with cord blood IgG ≤4 g/L experience significantly more culture-proven sepsis episodes than matched infants with IgG >4 g/L (P < 0.003) (42). Late-onset sepsis is in turn independently associated with moderate-to-severe BPD, as classified by the 2001 NICHD criteria (aOR 2.15, 95% CI 1.43–3.24), after adjustment for GA and other covariates (43), and systemic inflammation across the first two postnatal weeks is itself associated with BPD in infants born before the 28th week of gestation (44). These associations are best read as indirect: they run through susceptibility to infection and its attendant inflammatory hits rather than through any demonstrated direct effect of IgG concentration on alveolar development, and no study establishes that low IgG *per se* causes BPD.

## 5 Innate immune cell changes

Neutrophils are the earliest cellular responders to lung injury, recruited within hours of the initial insult. They release elastase, matrix metalloproteinases, ROS, and pro-inflammatory cytokines — each contributing to direct epithelial and endothelial damage. NETs have also been identified in tracheal aspirates of preterm infants (45).

Although multiple innate populations contribute to BPD, macrophages occupy a central position because they integrate upstream danger sensing with downstream inflammatory amplification and its resolution. During the canalicular-to-saccular transition, when the lung’s resident macrophage population is still being established, hyperoxia shifts the activation state of neonatal lung macrophages toward an M1-like, classically activated program, whose associated secretory function is production of IL-6, TNF-α, and GM-CSF (46–48); that state has been shown by transcriptomic analysis in newborn mice (46, 47) and by pro-inflammatory profiling of human airway monocytes (48). Cell number is a separate measurement: macrophage-derived markers and active STAT3 accompany the loss of alveolar epithelial cells in human BPD lung tissue (46), and transcriptomic analysis of hyperoxia-exposed neonatal mice identified downregulation of *Cybb* in M1 macrophages as the change most closely associated with macrophage infiltration; the same analysis inferred, but did not demonstrate, MIF-(CD74+CD44) signaling between AECs and macrophages (47). Within the monocyte compartment, CCR1+ monocytes are specifically upregulated in BPD and produce S100A8 and MMP8 via NF-κB and AKT pathways, serving as potential biomarkers and therapeutic targets (49). Airway CD14+ monocytes in BPD exhibit a pro-inflammatory transcriptomic profile with upregulated TLR9 expression and produce large amounts of GM-CSF, fueling a local positive-feedback inflammatory loop (48). Monocyte phenotype shifts (reflected by an increased Mon3:Mon1 ratio in peripheral blood) may additionally contribute to endothelial dysfunction characteristic of BPD (45).

M1 and M2 are used throughout this review as shorthand for the dominant activation program of a macrophage population rather than for two discrete cell types or for a count of cells. The M1/M2 classification is a useful conceptual framework, but it does not capture the full spectrum of macrophage activation states found *in vivo*; BPD macrophages likely occupy dynamic intermediate phenotypes that are influenced by the local cytokine milieu, oxygen tension, and developmental stage. Their metabolic programs are correspondingly flexible, engaging glycolytic and oxidative pathways together rather than partitioning cleanly by phenotype, and emerging human data indicate that neonatal lung macrophages follow a distinct developmental program that does not map neatly onto the adult M1/M2 axis (50).

The imprint of chronic M1 macrophage activation is written into the lung’s architecture: where the developmental program would normally elaborate thin-walled, richly vascularized gas-exchange units, the BPD lung presents instead with enlarged, simplified air spaces and thickened septal walls — a pattern documented in hyperoxia-exposed newborn mice and in BPD autopsy lung (46, 47). This cellular pathology is the biological substrate of the impaired gas exchange, pulmonary hypertension, and exercise intolerance that define the clinical syndrome and persist into adult life.

### 5.1 Additional innate immune effectors: NETs, innate lymphoid cells, dendritic cells, natural killer cells, and eosinophils

Beyond granule-mediated neutrophil injury and macrophage activation, five additional innate effector populations — NETs, type 2 innate lymphoid cells (ILC2s), dendritic cells, NK cells, and eosinophils — contribute distinct and intersecting mechanisms to BPD immunopathogenesis. Each is shaped by the developmental immaturity of the preterm immune system and each interacts with the TLR-macrophage inflammatory axis described above (**Figure 8**).

**Figure 8:**
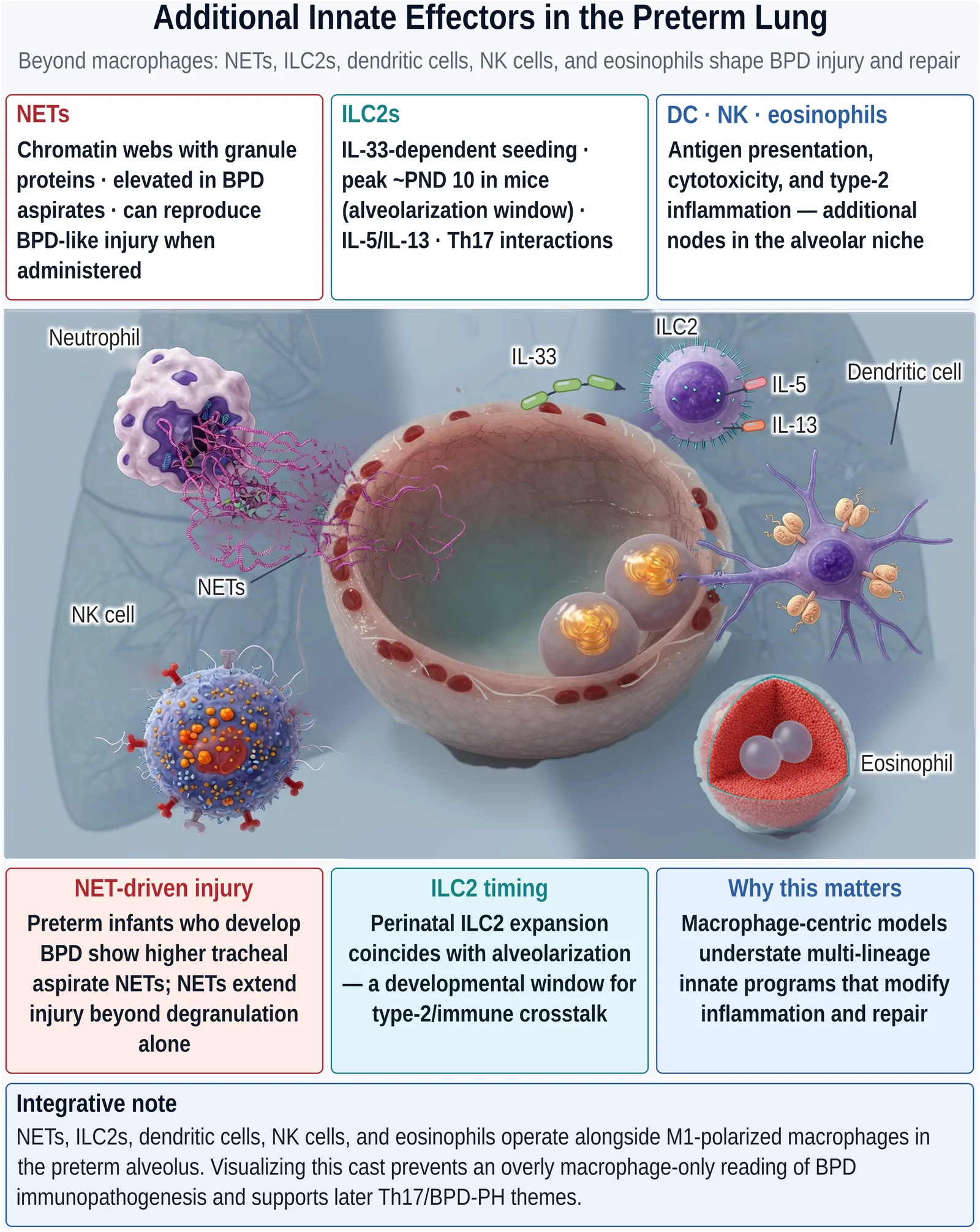
Additional innate effectors in the preterm lung. Beyond macrophages, neutrophil extracellular traps (NETs), group 2 innate lymphoid cells (ILC2s), dendritic cells (DCs), NK cells, and eosinophils contribute to BPD immunopathology. NETs are elevated in tracheal aspirates of infants who develop BPD and can reproduce BPD-like injury experimentally. ILC2s seed the lung under IL-33 influence and, in neonatal mice, peak near the alveolarization window. BPD, bronchopulmonary dysplasia; IL, interleukin; PND, postnatal day; Th, T-helper cells; DC, dendritic cell; NK, natural killer cell; PH, pulmonary hypertension.

#### 5.1.1 Neutrophil extracellular traps

NETs — decondensed chromatin studded with granule proteins — act as effectors of lung injury in BPD rather than as bystanders. Tracheal aspirate NET levels are higher in preterm infants who develop BPD, and intratracheal NETs reproduce alveolar simplification and reduce the alveolar differentiation markers AQP5 and SPC in neonatal mice (51). In hyperoxia-exposed neonatal rats, NET formation rises alongside histone release, and histones alone impair alveolarization and reduce VEGF (52). Two pathways carry the effect: NETs suppress WNT3a and β-catenin, downregulating c-MYC, cyclin D, and VEGF (51); and hyperoxia-injured alveolar epithelial cells *in vitro* recruit neutrophils through IL-8 released via adenosine A2A receptor-mediated ERK/p38 MAPK signaling on the IL-8/CXCR2 axis (53). Heparin and caffeine, the latter already standard of care, suppress NET formation and attenuate BPD-like injury in hyperoxia-exposed newborn rats (52) and in a newborn animal model of hyperoxia (53). Early aspirate NET levels (days 1–3) do not predict BPD, however, suggesting that NET-driven injury accumulates over the sustained inflammatory course rather than being present at its outset (45, 54).

#### 5.1.2 Innate lymphoid cells

ILC2s are seeded into the lung perinatally from fetal precursors in an IL-33-dependent manner (55, 56), peaking in neonatal mice around postnatal day 10, coincident with the alveolarization window (57). They arrive primed to produce IL-5 and IL-13, activated by a postnatal burst of IL-33 from type II alveolar epithelial cells (55, 57). Their role is dual. In hyperoxia-exposed neonatal mice, ILC2-driven IL-13 promotes epithelial-mesenchymal transition in type II AECs, and ILC2-derived AREG independently disrupts AEC II transdifferentiation through EGFR, both contributing to alveolar simplification; blocking type 2 cytokines reduces inflammation and improves function (58–61). ILC2s also carry reparative potential through effects on macrophage polarization and angiogenesis (58). In preterm infants, serum IL-33 correlates with IL-5 and IL-13 but not IL-4 — an ILC2-driven rather than classical Th2 signature — in a cohort compared with term-born infants rather than stratified by BPD severity (59); elevated serum IL-33, CCL24, and CCL13 in severe BPD are reported secondarily, with no severity definition given in the source (58). The activating node is the IL-33/ST2 axis, and the decoy receptor sST2 prevents asthma-like features in neonatal mice (55).

##### 5.1.3 Dendritic cells

DCs are present in the developing postcanalicular lung and increase more than three-fold with chorioamnionitis, mechanical ventilation, and BPD; their close association with pulmonary vessels implicates them in BPD dysangiogenesis (62). In neonatal mice, hyperoxia upregulates Flt3L, expanding CD103+ cDC1s (Batf3-dependent, Clec9a+) and pro-inflammatory CD11bhi subsets (63). Clec9a, a DAMP receptor for F-actin from necrotic airway cells, drives CD103+ DC production of IL-12p40; Batf3- and Clec9a-deficient mice show attenuated IL-12p40, IFN-γ, TNF-α, and airway hyperresponsiveness after viral challenge (64). In preterm tracheal aspirates, FLT3L is higher in infants who develop BPD and correlates with IL-12 and IFN-γ (63). Neonatal DCs are broadly immature, producing less IFN-α and IL-12p70 but equal or greater IL-1β, IL-6, IL-23, and IL-10 than adult cells — a profile favoring Th17/Th2 over Th1 polarization and enforced by chromatin-level repression of IL-12p35 (65–67); in neonatal mice, IL-33 further suppresses IL-12p35 while inducing OX40L, promoting Th2 skewing of CD4+ T cells (55). DCs therefore bridge innate danger sensing to the adaptive polarization that carries respiratory morbidity into later childhood.

##### 5.1.4 Natural killer cells

NK cells in preterm neonates are phenotypically immature: preterm cord blood shows fewer CD56bright NK cells and more inhibitory NKG2A+ cells, with other subsets comparable to term (68). Because CD56bright cells are the principal IFN-γ producers, antiviral function is expected to be diminished, though cytotoxicity was not measured directly (68, 69); IL-2 can nonetheless activate NK function as early as 20 weeks’ gestation, suggesting distinct maturation pathways for the two stimuli (70). Reduced NK percentage at birth is associated with late-onset infection in very preterm infants (71). In grade 2–3 BPD (Jensen 2019 criteria), NK frequencies are paradoxically increased and skewed toward cytotoxic CD56dimCD16+ cells expressing high granzyme B; whether this is compensatory or contributory is unresolved (72). Neonatal hyperoxia produces persistent change: exposed mice later challenged with influenza A show fewer reparative IL-22-producing and more cytotoxic IFN-γ-producing lung NK cells (73). Hyperoxia and endotoxin upregulate NKG2D ligands on stressed lung cells (discussed further in Section 6), and MICA and MICB are detectable in preterm tracheal aspirates, MICA correlating with FiO_2_ (74). Neonatal antibody-dependent cellular cytotoxicity is itself reduced relative to adult cells, and cord blood macrophages of preterm and term neonates express less CD16 (75, 76); because this effector arm requires antigen-specific IgG, deficient transplacental transfer would be expected to constrain it further, though that inference has not been tested directly in preterm infants (E4).

##### 5.1.5 Eosinophils

Eosinophils are less prominent than neutrophils in the acutely injured BPD airway but sustain the type 2 environment that shapes its long-term sequelae. The ILC2-derived IL-5 and IL-13 described above are also the principal eosinophilopoietic and eosinophil-activating cytokines, linking the IL-33/ST2 axis to eosinophil recruitment and survival (58, 59). The Th2-eosinophilic signature that predicts BPD severity from the first week of life reflects the same axis, and the asthma-like phenotype common among BPD survivors shows persistent type 2 airway inflammation with bronchodilator responsiveness (77). Whether eosinophils act as direct effectors of alveolar injury or principally as markers of a type 2 endotype remains unresolved; their consistent association with the asthma-like trajectory nonetheless makes IL-5/IL-13-directed strategies a rational consideration for that subset. A phenotype-wide association study found that SNPs associated with BPD also associate with mean eosinophil percentage and asthma-related phenotypes (78).

## 6 Adaptive immune cell changes

A growing body of evidence implicates adaptive immunity in BPD pathogenesis, with T helper cell imbalance emerging as a central theme. In hyperoxia-induced BPD models, Th17 cells are increased and regulatory T cells (Tregs) are decreased, resulting in an elevated Th17/Treg ratio, rising lung IL-17A levels, and declining anti-inflammatory IL-10 (79). IL-17A-producing γδ T cells activated through NKG2D signaling are key drivers of endotoxin-induced neonatal lung injury, and tracheal aspirates from preterm infants who later develop BPD show higher levels of both IL-17A and its upstream regulator IL-23 (74) (**Figure 9A**).

**Figure 9:**
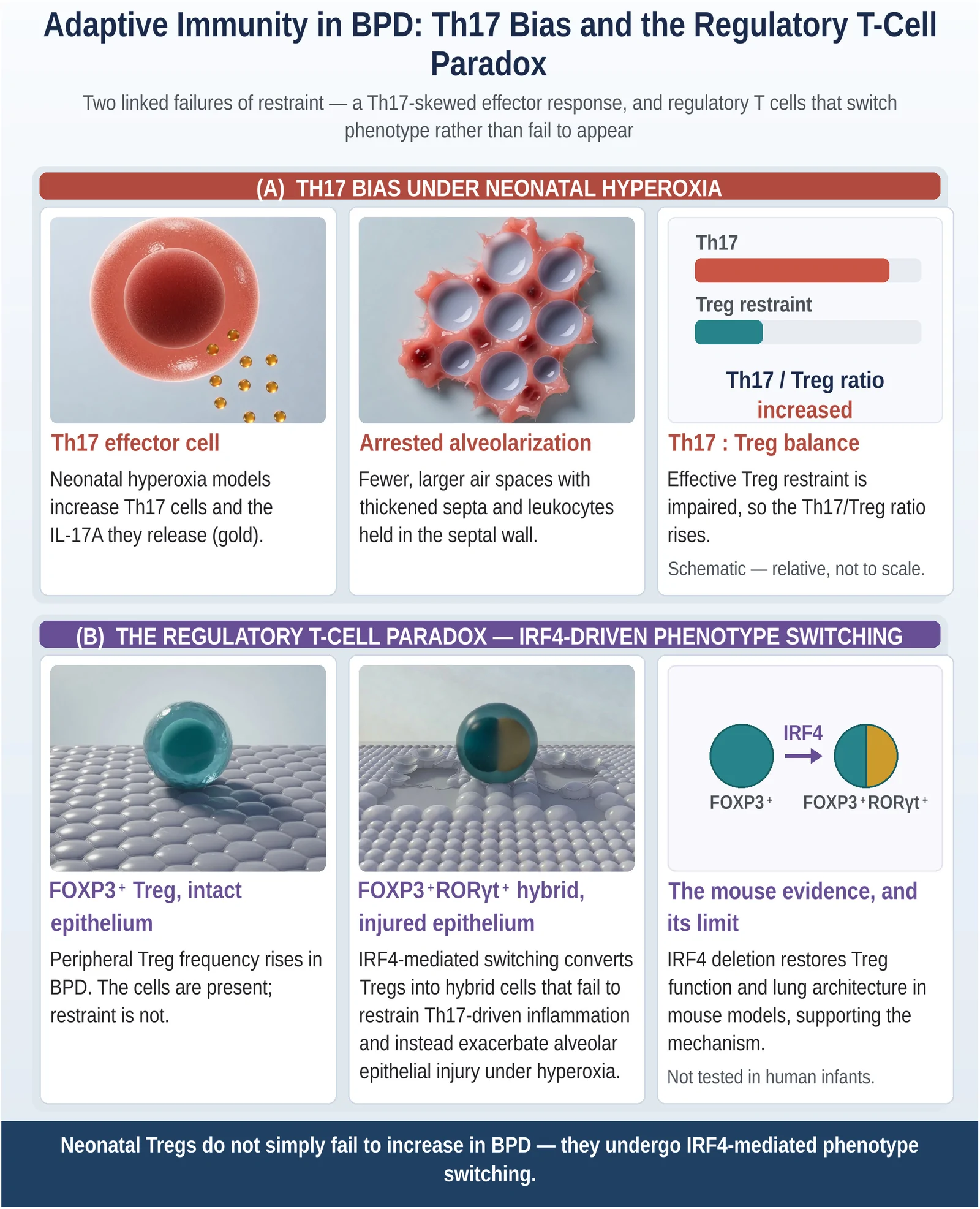
Adaptive immunity in BPD: Th17 bias and the regulatory T-cell paradox. **(A)** Neonatal hyperoxia models increase Th17 cells and lung IL-17A while impairing effective Treg restraint, elevating the Th17/Treg ratio. **(B)** Neonatal Tregs do not simply fail to increase in BPD; they rise in peripheral frequency yet undergo IRF4-mediated phenotype switching, converting FOXP3+ Tregs into hybrid FOXP3+RORγt+ cells that fail to restrain Th17-driven inflammation and instead actively exacerbate alveolar epithelial injury under hyperoxia; in mouse models *IRF4* deletion restores Treg function and lung architecture, supporting the mechanism, which has not been tested in human infants. BPD, bronchopulmonary dysplasia; Th17, T helper 17; Treg, regulatory T cell; IL-17A, interleukin-17A; FOXP3, forkhead box P3; RORγt, retinoic acid receptor-related orphan receptor gamma t; IRF4, interferon regulatory factor 4.

Perhaps the most counterintuitive finding in BPD immunology is that the very cells tasked with dampening inflammation appear to contribute to it. Increased Treg frequencies in peripheral blood have been observed to precede BPD development in preterm infants — a finding that inverts the expected relationship between immune regulation and disease protection (80). These neonatal Tregs are not simply more numerous than those of term infants or adults; they are phenotypically distinct and by day 7 of life transition to a highly activated state with suppressive capacity that surpasses anything observed in the mature immune system (baseline phenotype: CD45RA+/HLA-DR−/Helios+; by day 7: CCR6+, HLA-DR+, Ki-67+) (80).

This paradox — that a cell population defined by its ability to suppress inflammation may simultaneously sustain it — likely reflects a fundamental property of the neonatal immune system: in a preterm infant, whose lungs are still being colonized by the microbial exposures of extrauterine life, excessive suppression of adaptive immunity may impair pathogen clearance, allowing persistent microbial stimulation that perpetuates TLR-driven cytokine production even as acquired immune responses are dampened. The result is an immune environment that is simultaneously over-suppressed and over-inflamed, with each arm reinforcing the other. Compounding this complexity, in hyperoxia-exposed neonatal mice IRF4-mediated Treg phenotype switching (in which FOXP3+ Tregs convert to FOXP3+RORγt+ cells) exacerbated AEC injury, while *IRF4* deletion restored normal Treg function and lung architecture (**Figure 9B**) (81, 82). Lower peripheral blood CD4+ T cell ratios correlate with grade 2–3 BPD in the same cohort, reflecting consumption of naive T cells by early immune activation (72).

Th2-mediated responses are also relevant in BPD survivors, particularly in the context of respiratory viral infections such as RSV and rhinovirus. These infections contribute to airway remodeling and obstructive lung disease through Wnt/β-catenin pathway activation, underscoring the long-term immunological consequences of early-life lung injury in this population (83).

## 7 Key cytokines and soluble mediators

The cytokine environment in the BPD lung is not simply elevated — it is qualitatively transformed. From the first hours of life, a self-amplifying network of pro-inflammatory mediators spanning multiple classes accompanies, and is inferred to sustain, the macrophage activation, neutrophil recruitment, endothelial injury, and fibroblast proliferation that collectively impair normal lung development (3, 84). Understanding this network is essential for identifying which nodes are most tractable as therapeutic targets.

IL-1β is detectable from day 1 in tracheal aspirates and in murine lavage fluid, and the IL-1β:IL-1Ra ratio in tracheal aspirates predicts death or BPD at 36 weeks (3, 4). IL-6, elevated in both tracheal aspirates and serum, exerts its most damaging effects through trans-signaling via STAT3, impairing alveolar type II cell survival and disrupting elastic fiber assembly. Elevated IL-6 is a high-risk factor for BPD (46), particularly at days 7–14 and 21–28 of life (84). IL-8, a potent neutrophil chemoattractant, drives neutrophil infiltration (3); serum IL-8 predicts BPD (84).

TNF-α becomes a significant risk factor by days 21–28 of life, inducing fibroblast proliferation and collagen production; single nucleotide polymorphisms in TNF-α predict both BPD onset and severity (3, 84). IL-17A is elevated in tracheal aspirates of infants who develop BPD, though its human airway source is undetermined; in rodent models both γδ T cells and Th17 cells contribute (74, 79). IL-10 is anti-inflammatory and appears protective in animal models (79); lower serum IL-10 is consistently associated with BPD development (84). Leukotrienes are elevated in BPD lungs and remain raised at 6 months of age, causing bronchoconstriction, vasoconstriction, edema, and mucus production, while sICAM-1 is elevated from day 1, promoting cell-to-cell contact and further cytokine production (3).

Taken together, this cytokine network illustrates why BPD is not simply an inflammatory state but an inflammatory failure state: the mediators that should resolve as the lung matures instead amplify and reinforce each other, with IL-1β and IL-6 sustaining macrophage and neutrophil recruitment, TNF-α promoting fibroblast proliferation, and the deficit of IL-10 removing the principal brake on this cascade. This is not simply a network that fails to switch off; it is a network that reorganizes into a new, self-sustaining configuration, in which mediators that would normally operate in sequence — initiation, amplification, resolution — instead operate in a persistent feed-forward loop. The practical corollary is that no single cytokine antagonist is likely to be sufficient; effective therapeutic strategies will need to either interrupt the upstream signaling nodes that drive the entire network (such as TLR4 or the NLRP3 inflammasome) or broadly reprogram the macrophage polarization state that is the network’s primary cellular source.

### 7.1 Specialized pro-resolving mediators: the endogenous resolution system and its failure in BPD

If inflammatory initiation explains how BPD begins, the failure of SPM biology explains why it persists. That program is executed by the SPMs: a structurally diverse family of bioactive lipid mediators biosynthesized from PUFA precursors that actively terminate inflammation, clear apoptotic debris, and restore tissue homeostasis. The four principal SPM families are lipoxins (from arachidonic acid), resolvins (from DHA and EPA), protectins (from DHA), and maresins (from DHA via macrophage 12-LOX), each acting through distinct G-protein-coupled receptors (FPR2/ALX, GPR18, GPR32) to coordinate neutrophil egress, macrophage efferocytosis, and anti-inflammatory gene transcription (8, 9, 85, 86).

The biosynthetic pathways generating these mediators are exquisitely susceptible to disruption in the preterm lung. Lipoxin A4 synthesis requires sequential oxygenation of arachidonic acid by 15-LOX (generating 15S-HETE) followed by 5-LOX (generating LXA4), or alternatively, transcellular platelet 12-LOX acting on neutrophil-derived LTA4. Resolvin D1, protectin D1, and maresins all require DHA as their primary substrate, liberated from membrane phospholipids by phospholipase A2 (87) and converted by 15-LOX to 17S-H(p)DHA and then routed through distinct epoxidation and hydrolysis steps by 5-LOX or 12-LOX (8, 9). Multiple steps in this cascade are compromised in the preterm infant. DHA and arachidonic acid stores are established primarily during the third trimester through transplacental transfer; infants born before 28 weeks arrive with severely depleted PUFA reserves, and these decline further in the first postnatal week regardless of enteral feeding (88). Beyond substrate deficiency, 5-LOX enzymatic behavior under inflammatory conditions favors pro-inflammatory product generation: nuclear membrane-localized 5-LOX (the configuration promoted by TLR4 activation and impaired efferocytosis signaling) preferentially generates leukotriene B4 (LTB4) rather than LXA4, while cytoplasmic localization (driven by MerTK efferocytosis signaling) favors LXA4 — a regulatory geometry established in adult human monocyte-derived macrophages, murine peritonitis, and atherosclerotic lesions rather than demonstrated directly in the preterm lung, where alveolar macrophages are transcriptionally immature and this localization has not been shown (89–91). In the neonatal hyperoxic lung, where MerTK expression on alveolar macrophages is developmentally reduced, 5-LOX remains nuclear-membrane-associated, shunting PUFA metabolism toward LTB4 and away from LXA4 (**Figure 10**). Direct lipidomic evidence from tracheal aspirates of extremely preterm infants confirms this pattern: an untargeted lipidomic analysis identified 298 significantly different lipid metabolites between BPD and non-BPD infants at intubation, with notable depletion of phosphatidylcholine and phosphatidylethanolamine membrane pools (the substrate reservoirs from which PLA2 liberates SPM precursors) and accumulation of ceramides and triglycerides in the BPD group (92).

**Figure 10:**
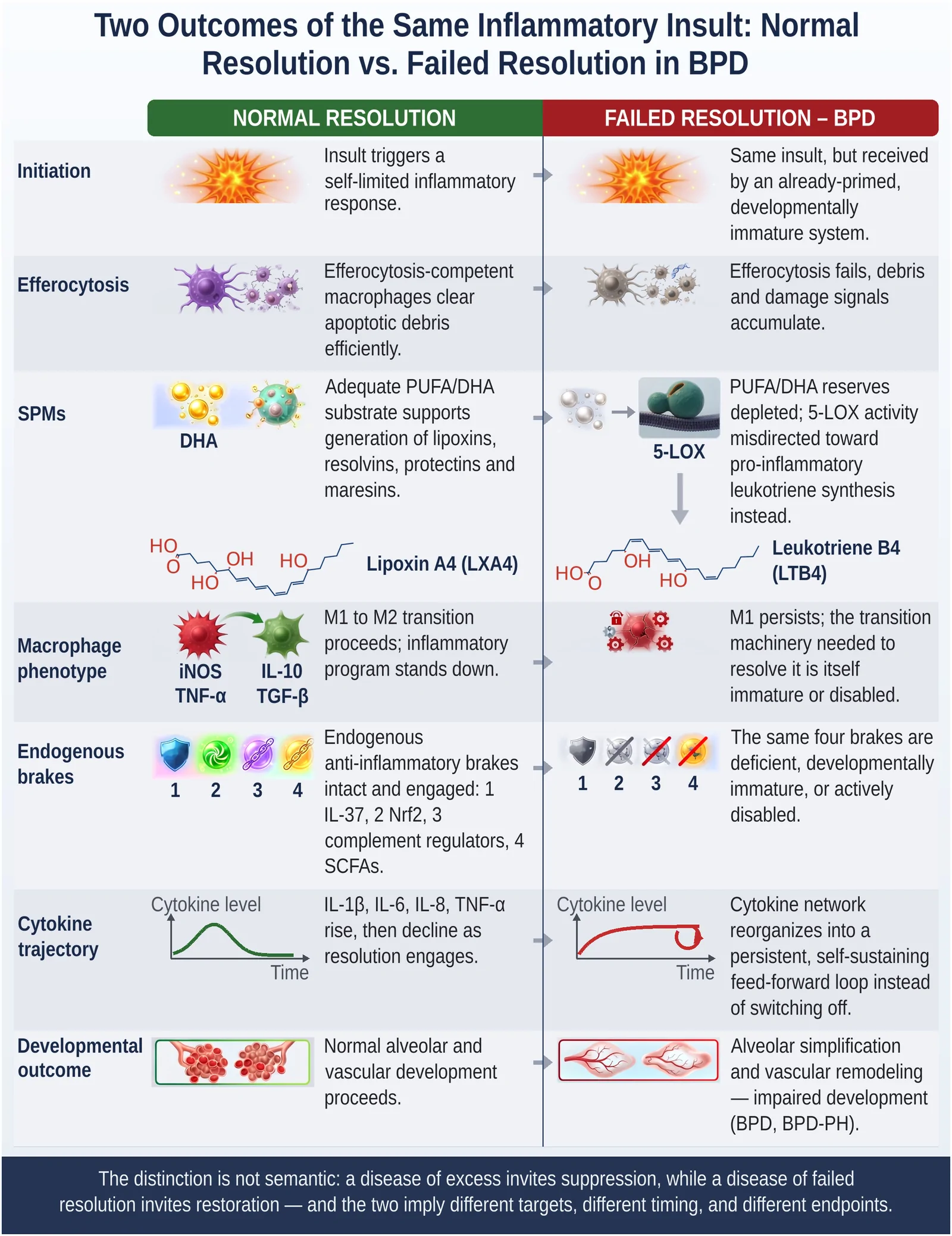
Two outcomes of the same inflammatory insult: normal resolution vs. failed resolution in BPD. Side-by-side comparison of seven sequential steps — initiation, efferocytosis, specialized pro-resolving mediator (SPM) biosynthesis, macrophage phenotype, endogenous anti-inflammatory brakes (IL-37, Nrf2, complement regulators, short-chain fatty acids), cytokine trajectory, and developmental outcome — contrasting the self-limited course of normal inflammatory resolution with the persistent, self-sustaining feed-forward loop that characterizes failed resolution in BPD. In the macrophage phenotype row, M1 and M2 denote dominant activation programs; “M1 persists” describes a failure of that program to shift rather than a fixed population of cells. SPM, specialized pro-resolving mediator; PUFA, polyunsaturated fatty acid; DHA, docosahexaenoic acid; LOX, lipoxygenase; iNOS, inducible nitric oxide synthase; IL, interleukin; TNF, tumor necrosis factor; TGF, transforming growth factor; BPD, bronchopulmonary dysplasia; PH, pulmonary hypertension.

The downstream consequences of SPM deficiency would plausibly create a self-amplifying cycle capable of accounting for the sustained macrophage activation that characterizes established BPD. Three interconnected mechanisms are operative. First, where LXA4 and RvE1 are deficient, neutrophil clearance is impaired, and by extension from adult lung disease, neutrophils persist in the alveolar space and continue to release proteases and ROS against the developing epithelium (9, 86). Second, macrophage efferocytosis — the clearance of apoptotic neutrophils that normally terminates inflammatory signaling — is enhanced *in vitro* by nearly all SPMs; conversely, MerTK signaling drives the cytoplasmic 5-LOX translocation and LXA4 synthesis that sustain SPM supply, so efferocytosis failure and SPM deficiency are mutually reinforcing (89, 90, 93, 94). Third, RvD2 directly suppresses the NLRP3 inflammasome by inhibiting ASC oligomerization and caspase-1 activation via GPR18 signaling, reducing mature IL-1β secretion; in its absence, the NLRP3 axis (already primed by hyperoxia and TLR4 activation) operates without this endogenous brake (95). The consequence at the macrophage level is maintained M1 polarization with sustained NF-κB-driven IL-1β and TNF-α production. In neonatal rats, LXA4 not only promotes M2 polarization but also drives M2-derived exosomal miR-25-5p release, which protects alveolar type II cells from pyroptosis through the NRBP2/PI3K/AKT axis — linking SPM supply to alveolar epithelial survival (96).

The resolution program of the preterm lung fails at four separable points (substrate depletion, TLR4-driven nuclear-membrane 5-LOX localization, efferocytosis failure, and loss of resolvin D2-mediated NLRP3 restraint), and a defect at any one of them would in principle be sufficient to convert a self-limited inflammatory response into a sustained one, though sufficiency has not been tested experimentally at any single point; the two divergent outcomes — normal resolution versus the failed-resolution loop of BPD — are contrasted step by step in **Figure 10**. Direct measurement of the mediators themselves remains the most important descriptive gap in this area: we identified no human study quantifying lipoxin A4, resolvin D1, protectin D1, or maresin 1 in the preterm airway as a function of GA, lipid intake, or BPD outcome. The existing human evidence is inferential, resting on lipidomic depletion of the membrane phospholipid pools from which these mediators are liberated (92).

### 7.2 IL-37: anti-inflammatory mechanisms and NLRP3 inflammasome suppression

IL-37 is an emerging candidate pathway in BPD. No published study has measured IL-37 in preterm tracheal aspirates, cord blood, or plasma comparing infants who develop BPD with those who do not, and neither IL-37 nor its co-receptors have been mapped across the developing human lung. The supporting evidence is correspondingly indirect: transfected cell lines and human macrophage cultures, transgenic mice, and adult, largely non-pulmonary disease models, together with a single neonatal lung-injury study and no hyperoxia-based model with alveolarization endpoints. The evidence is therefore E3 and E4 in the Section 2 scheme. It is considered here because IL-37’s target set matches the pathology described above with unusual precision.

IL-37, formerly designated IL-1F7, is the principal IL-1 superfamily member that acts as a broad anti-inflammatory agonist cytokine rather than a pro-inflammatory signal, distinct from IL-1Ra, which acts by competitive receptor blockade. Of the five splice variants encoded in the IL-1 gene cluster on chromosome 2q12-13, IL-37b is the most abundant and most extensively studied and is the isoform used in transgenic models; IL-37a and IL-37d also form the functional β-trefoil structure, and, in mouse macrophages, IL-37d suppresses NLRP3 transcription (97–100).

IL-37 signals through two complementary routes, set out in full in **Figure 11**. Extracellularly it binds the IL-18 receptor α chain (IL-18Rα) and recruits the orphan receptor IL-1R8 (SIGIRR/TIR8), forming a tripartite complex that sequesters MyD88 and activates anti-inflammatory rather than pro-inflammatory cascades; IL-1R8-deficient IL-37 transgenic mice lose protection from endotoxemia, and both receptors are required for IL-37 activity (97, 99, 101, 102). Intracellularly, caspase-1 cleaves the precursor at Asp20 and mature IL-37 translocates with phosphorylated Smad3 to repress pro-inflammatory transcription (103–105).

**Figure 11:**
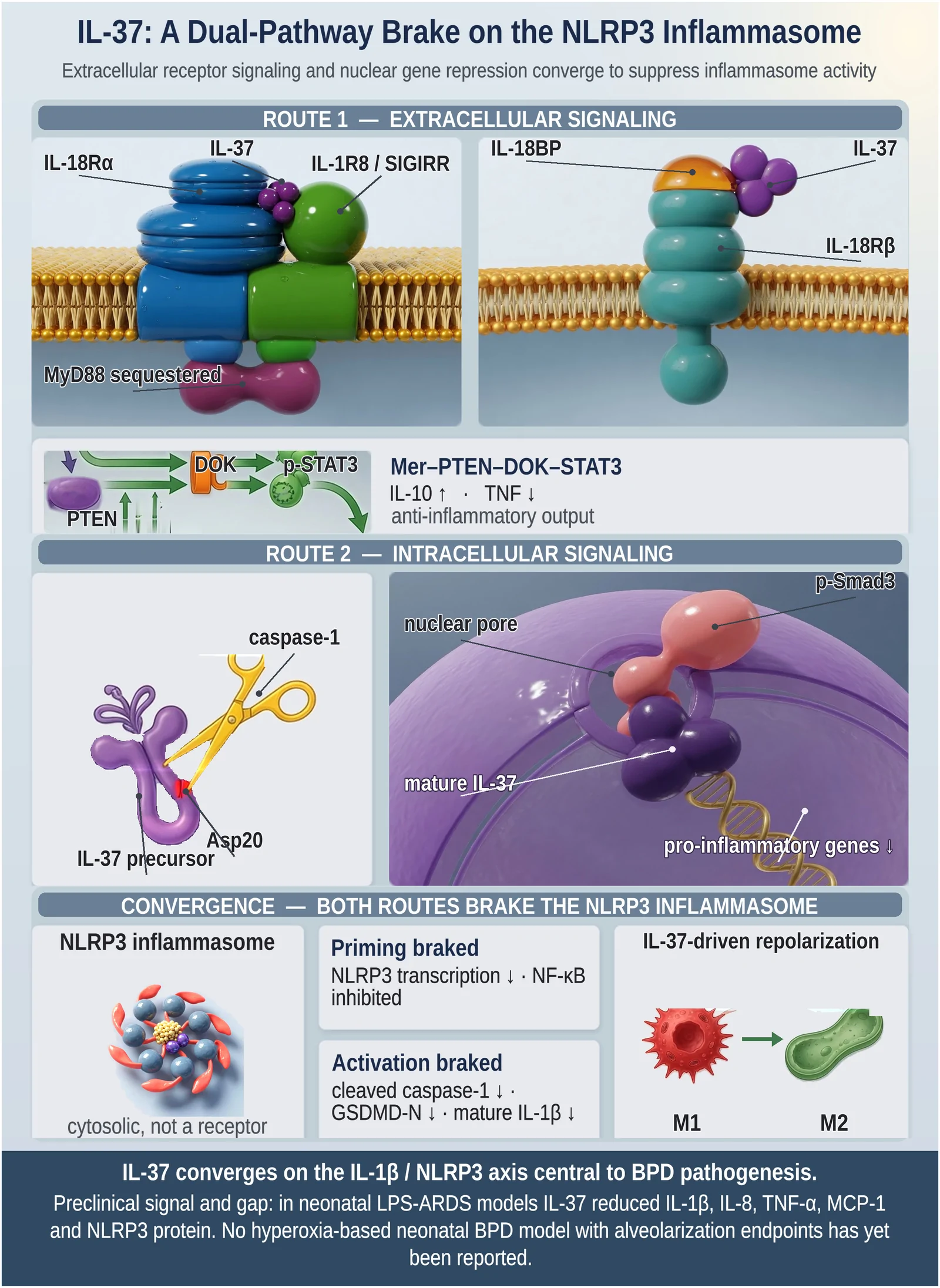
IL-37 dual-pathway anti-inflammatory signaling and NLRP3 inflammasome suppression. IL-37, the principal broadly anti-inflammatory member of the IL-1 family, acts through two distinct routes. It is distinct from IL-1Ra, which is anti-inflammatory but acts by competitive receptor blockade rather than as an agonist cytokine, and from IL-38, which also exerts context-dependent anti-inflammatory effects. Extracellularly, secreted IL-37 binds IL-18Rα and recruits the orphan receptor IL-1R8 (SIGIRR), forming a tripartite complex that sequesters MyD88 and activates anti-inflammatory Mer–PTEN–DOK and STAT3 cascades; IL-37 also sequesters IL-18Rβ through IL-18 binding protein. Intracellularly, caspase-1 cleaves the IL-37 precursor at Asp20, and mature IL-37 binds phospho-Smad3 and translocates to the nucleus to repress pro-inflammatory gene transcription. Both routes converge on NLRP3 inflammasome suppression at the priming step (reduced NLRP3 transcription via NF-κB inhibition) and the activation step (reduced cleaved caspase-1, gasdermin D-N, and mature IL-1β), driving M1-to-M2 macrophage repolarization; repolarization denotes a shift in the dominant activation program rather than conversion between two discrete cell types. Convergence with the IL-1β/NLRP3 axis central to BPD makes IL-37 a therapeutic candidate, although no hyperoxia-based neonatal model with alveolarization endpoints has yet been reported. IL-18Rα/β, interleukin-18 receptor α/β chain; IL-1R8, interleukin-1 receptor 8 (SIGIRR/TIR8); GSDMD-N, gasdermin D N-terminal fragment; NLRP3, NOD-, LRR- and pyrin domain-containing protein 3; p-Smad3, phosphorylated Smad3; NF-κB, nuclear factor kappa B; TNF, tumor necrosis factor; PTEN, phosphatase and tensin homolog; ASC, apoptosis-associated speck-like protein containing a CARD (caspase recruitment domain); iNOS, inducible nitric oxide synthase; LPS, lipopolysaccharide; ARDS, acute (adult) respiratory distress syndrome; MCP, monocyte chemoattractant protein.

The breadth of IL-37’s anti-inflammatory output is striking, and almost all of it has been established *in vitro*: in LPS-stimulated macrophages IL-37 suppresses IL-1β, TNF-α, IL-6, IL-12, IL-8, IL-23, IFN-γ, and MIP-2/CXCL2, with recombinant IL-37 most effective at picomolar concentrations in M1-polarized human macrophages — the phenotype that dominates the BPD lung (99, 104, 106). Its convergence with the NLRP3 inflammasome (Section 8.1) operates at both steps: at priming, IL-37d suppresses NLRP3 expression through IL-1R8-dependent NF-κB inhibition (100); at activation, IL-37 reduces cleaved caspase-1, cleaved IL-1β, and gasdermin D in mouse macrophages (107). The dependence runs both ways, since IL-37 transfected into ASC- or NLRP3-deficient macrophages fails to suppress IL-6 or IL-1β (105). The *in vivo* evidence, however, is adult and largely non-pulmonary — alum-induced peritonitis, DSS colitis, periodontitis, and ventilator-induced lung injury (100, 107, 108). The one exception is directly relevant here: IL-37 attenuated inflammation, apoptosis, and NLRP3 expression in LPS-induced neonatal acute respiratory distress syndrome in six-day-old mice, the only neonatal lung model in which IL-37 has been tested, and one whose endpoints were inflammatory rather than structural (109). In these models and in human monocyte lines the recurring signature is a shift from M1 to M2 macrophage polarization, through NLRP3 or NF-κB/Notch1 inhibition (110–112).

Two features nonetheless situate IL-37 within BPD rather than within inflammation generally. The cytokine profile it opposes is the profile that characterizes infants who develop BPD — IL-6, IL-8, TNF-α, and IL-1β elevated in tracheal aspirates and plasma from the first postnatal days, with anti-inflammatory IL-10 insufficient to restrain them — and IL-37 has been proposed specifically as a candidate mediator of the neonatal vascular inflammation that accompanies it (113–116). Its point of action is also the node this review identifies as central. A relative IL-37 deficiency in BPD remains a plausible but untested hypothesis.

## 8 Key signaling pathways

NF-κB serves as the master transcriptional regulator of inflammation in BPD, activated by cytokines, LPS (via TLR4), and oxidative stress. Its downstream targets include TNF-α, IL-1, IL-6, IL-8, ICAM-1, iNOS, and COX-2 (2). Closely linked to this pathway, NLRP3 inflammasome activation is critically involved in BPD development: hyperoxia activates the NLRP3 inflammasome, leading to caspase-1 activation and secretion of mature IL-1β and IL-18. The genetic evidence that NLRP3-knockout mice exposed to hyperoxia are protected from alveolar simplification — establishing NLRP3 as necessary and non-redundant in these neonatal hyperoxia models — is detailed in Section 8.1 (4). In a neonatal rat model, dexamethasone counteracts NLRP3-induced pyroptosis through the SIRT1/NF-κB pathway (117). The TGF-β and angiogenic developmental signaling cascades (essential for alveolar septation and pulmonary angiogenesis) are disrupted by sustained inflammation in newborn transgenic mice, providing a mechanistic link between immune activation and the impaired lung development that defines BPD (2, 118, 119).

### 8.1 The NLRP3 inflammasome in BPD

The NLRP3 inflammasome occupies a central position in BPD immunopathogenesis that warrants dedicated consideration. Direct evidence for this pathway in BPD is derived primarily from rodent hyperoxia models; corroborating data in human infants remain limited. It functions as a cytoplasmic surveillance complex: upon sensing danger signals (including mitochondrial ROS, ATP release from necrotic cells, and uric acid crystals generated during hyperoxia), NLRP3 recruits the adaptor protein ASC and pro-caspase-1. Caspase-1 activation then cleaves pro-IL-1β and pro-IL-18 into their mature, secreted forms and cleaves GSDMD to generate GSDMD-N-terminal pores in the plasma membrane, releasing cytokines and triggering pyroptotic cell death (2, 4) (**Figure 12**).

**Figure 12:**
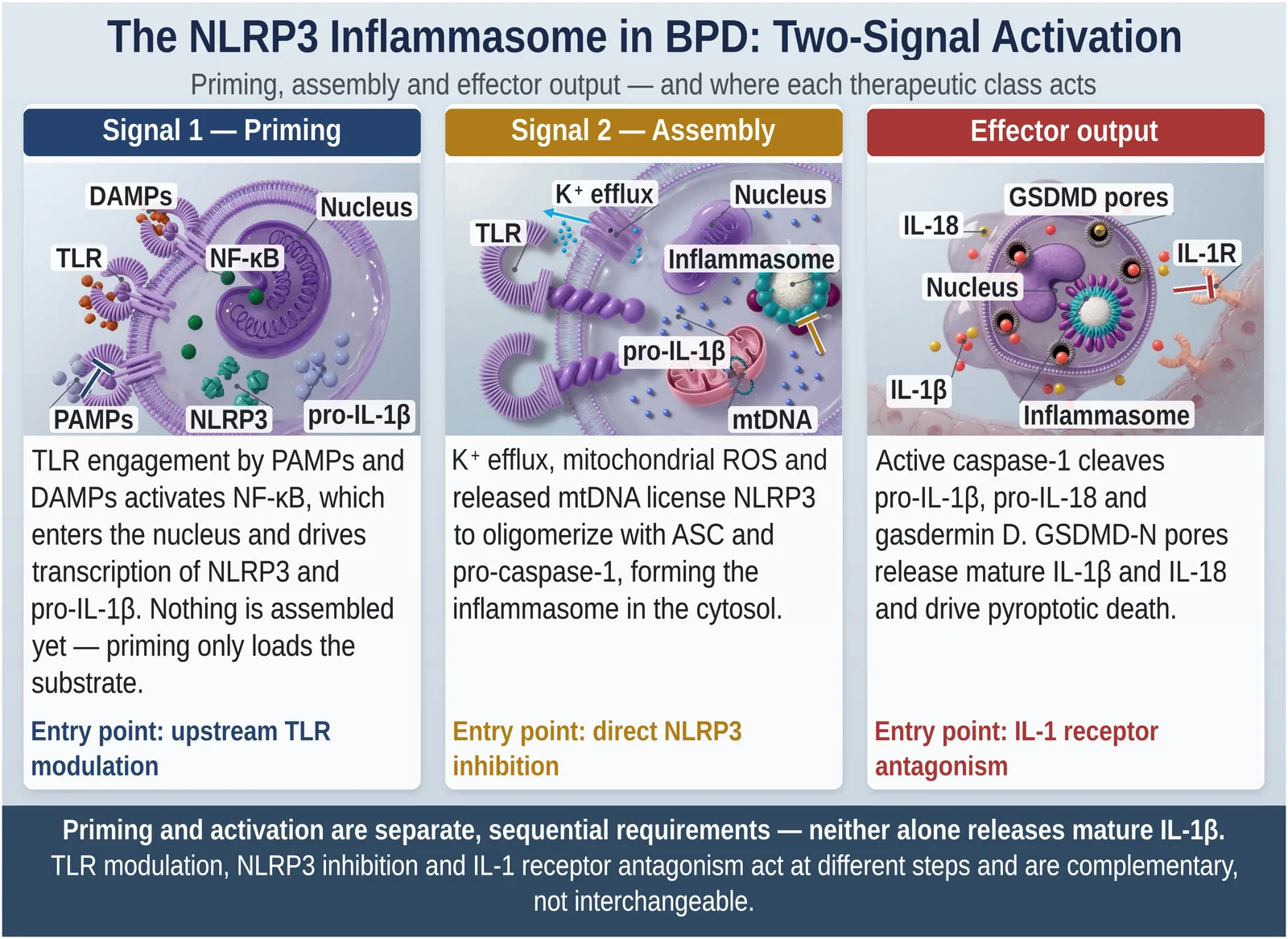
The NLRP3 inflammasome in BPD: two-signal activation. Signal 1 (priming) via TLR/NF-κB induces NLRP3 and pro-IL-1β transcription. Signal 2 (activation) via K+ efflux, ROS, and mitochondrial DAMPs assembles the NLRP3–ASC–caspase-1 complex, generating mature IL-1β/IL-18 and gasdermin-D-dependent pyroptosis. Therapeutic entry points include upstream TLR modulation, direct NLRP3 inhibition, and IL-1 receptor antagonism. NLRP3, NOD-, LRR- and pyrin domain-containing protein 3; ASC, apoptosis-associated speck-like protein containing a CARD (caspase recruitment domain); GSDMD, gasdermin D; TLR, Toll-like receptor; NF-κB, nuclear factor kappa B; IL, interleukin; ROS, reactive oxygen species; mtDNA, mitochondrial DNA; DAMPs, damage-associated molecular patterns; PAMPs, pathogen-associated molecular patterns.

The NLRP3 inflammasome is activated in the neonatal lung under hyperoxic conditions through multiple converging signals, and its pathological consequences are severe: NLRP3-knockout neonatal mice exposed to hyperoxia show no caspase-1 activation, no mature IL-1β production, and undergo normal alveolarization — establishing NLRP3 as necessary and non-redundant for alveolar simplification in this neonatal hyperoxia model; whether the same necessity holds in human preterm lung disease, where the evidence remains associative, is not established (4). NLRP3 activation in the lung is not isolated: hyperoxia simultaneously activates the NLRP1 inflammasome in brain tissue, and alveolar macrophage-derived extracellular vesicles carrying the ASC adaptor, when transferred into newborn mice, cross the blood-brain barrier and induce hippocampal neuroinflammation and pyroptosis (120). This shared inflammasome-driven pathobiology across lung and brain identifies the inflammasome as a candidate target for interventions designed to simultaneously protect both organs.

NLRP3 activation in BPD is intimately coupled to TLR4 signaling through the TLR4–CD44–NLRP3 axis: TLR4 activation by hyperoxia-generated DAMPs upregulates CD44, which acts as a co-receptor to amplify NLRP3 priming signals, creating a self-reinforcing loop in which each round of tissue injury deepens NLRP3 activation. The Nrf2 antioxidant pathway (Section 8.2) acts as a brake on this cycle by reducing the ROS that prime the NLRP3 complex, and in neonatal mice Nrf2 deficiency worsens NLRP3-mediated pyroptosis and impairs alveolar growth (121). Pharmacological approaches targeting the NLRP3 pathway include glyburide (a sulfonylurea that inhibits NLRP3 but is not a selective NLRP3 inhibitor, with additional actions on SUR1/KATP channels), dexamethasone (SIRT1/NF-κB-mediated pyroptosis suppression), and tert-butylhydroquinone (Nrf2/HO-1-mediated ROS suppression), none of which are evaluated here.

### 8.2 Additional key signaling mechanisms: Nrf2/antioxidant defense and trained immunity

Two additional signaling mechanisms intersect with the NF-κB, NLRP3, TLR2/TLR4, and developmental growth factor pathways described above: the Nrf2-mediated antioxidant response and trained immunity — the epigenetic reprogramming of innate myeloid cells by prior inflammatory exposures. Each represents an under-recognized dimension of BPD immunopathogenesis with emerging therapeutic implications.

#### 8.2.1 Nrf2 and the antioxidant response

Nrf2 is the master transcriptional activator of the antioxidant response. Much of this evidence derives from Nrf2-deficient mouse models; the extent to which this pathway is dysregulated in human preterm infants is less well characterized. Under basal conditions, Nrf2 is sequestered in the cytoplasm by its inhibitor Keap1, which targets it for proteasomal degradation. Upon oxidative stress (as occurs during supplemental oxygen therapy in preterm infants), reactive electrophiles modify critical cysteine residues on Keap1, releasing Nrf2 to translocate to the nucleus and bind AREs in the promoters of cytoprotective genes (122, 123). During saccular lung maturation, Nrf2 modulates genes involved in redox balance, lung morphogenesis, cell growth, and immunity, and Nrf2-deficient newborn mice exposed to hyperoxia exhibit increased mortality and more severe alveolar growth inhibition than wild-type counterparts (122). Conversely, hyperoxia-exposed premature rats show upregulation of both Nrf2 and HO-1, reflecting an endogenous but ultimately insufficient compensatory response (124). The most direct evidence for Nrf2 insufficiency in BPD comes from neonatal mice bearing hypomorphic Keap1 alleles, in which constitutively elevated Nrf2 reduced hyperoxia-induced hypoalveolarization from 28.9% to 2.4%, accompanied by increased antioxidant gene expression and glutathione (GSH) levels (125) (**Figure 13**).

**Figure 13:**
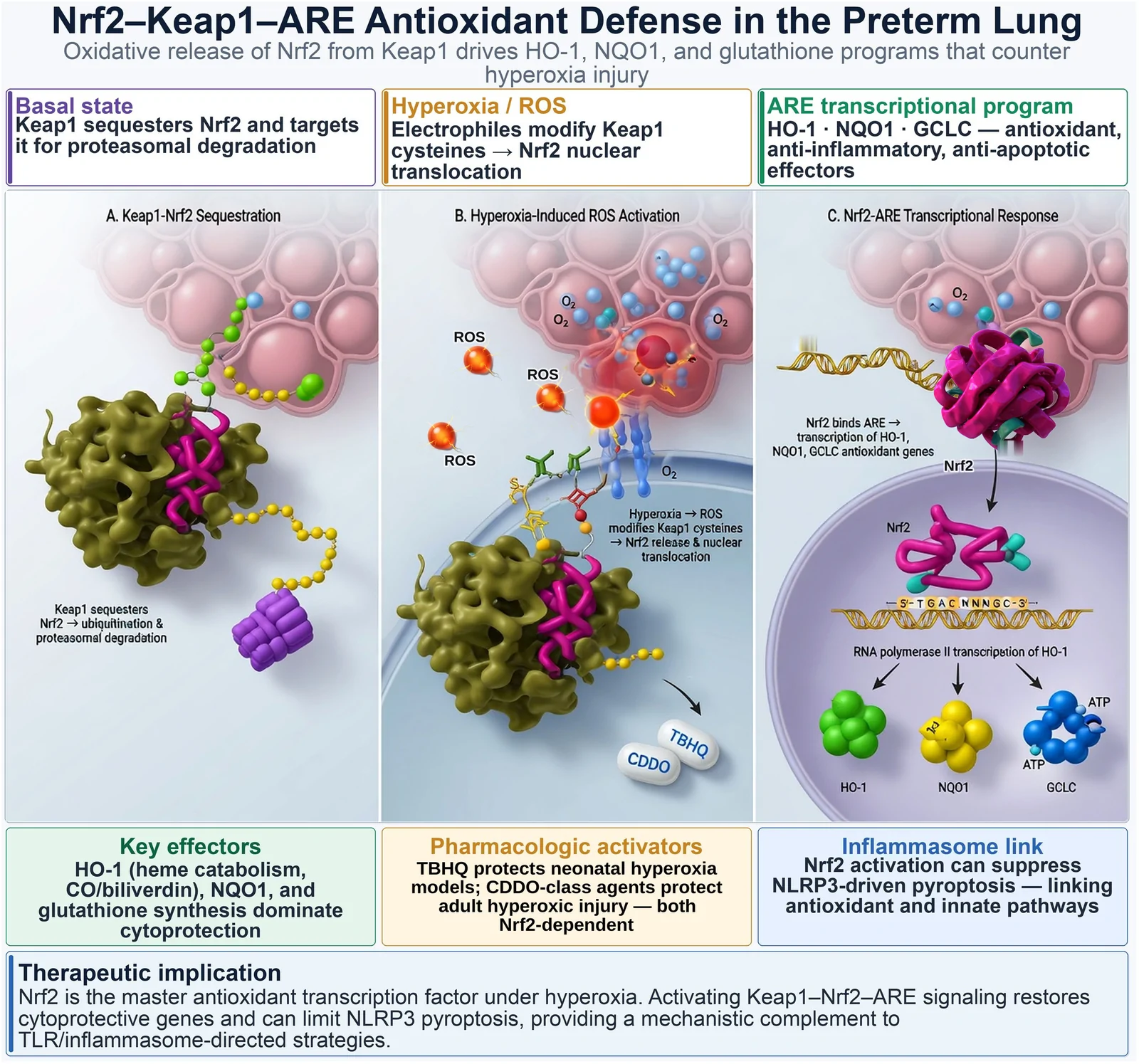
Nrf2–Keap1–ARE antioxidant defense in the preterm lung. Under basal conditions Keap1 targets Nrf2 for degradation; hyperoxia-derived electrophiles release Nrf2 for nuclear antioxidant-response-element transcription (HO-1, NQO1, GCLC). Pharmacologic Nrf2 activators protect neonatal hyperoxia models (TBHQ) and adult hyperoxic lung injury (CDDO-imidazolide) in an Nrf2-dependent manner, and can limit NLRP3-linked pyroptosis. Panels: **(A)** basal Keap1–Nrf2 sequestration; **(B)** hyperoxia-induced ROS activation; **(C)** the Nrf2–ARE transcriptional response. Nrf2, nuclear factor erythroid 2-related factor 2; Keap1, Kelch-like ECH-associated protein 1; ARE, antioxidant response element; HO-1, heme oxygenase-1; NQO1, NAD(P)H quinone dehydrogenase 1; ROS, reactive oxygen species; CO, carbon monoxide; GCLC, glutamate-cysteine ligase catalytic subunit; TBHQ, tert-butylhydroquinone; NLRP3, NOD-, LRR- and pyrin domain-containing protein 3; CDDO, 2-cyano-3,12-dioxooleana-1,9(11)-dien-28-oic acid; TLR, Toll-like receptor.

The principal Nrf2-regulated effectors relevant to BPD are HO-1, NQO1, and GCLC. HO-1 catalyzes heme degradation to biliverdin, carbon monoxide, and free iron, exerting antioxidant, anti-inflammatory, and anti-apoptotic effects; the Nrf2/HO-1 axis is the most extensively studied cytoprotective arm in the preterm lung and is considered more potent than activation of any single antioxidant gene in isolation (123). NQO1 protects against hyperoxia-induced apoptosis in lung epithelial cells (siRNA knockdown of Nrf2 significantly downregulates NQO1 and increases apoptosis) (126) while GCLC, the rate-limiting enzyme in GSH biosynthesis, is a canonical Nrf2 target whose pathway disruption is confirmed by integrated transcriptomic-metabolomic analysis of hyperoxia-exposed neonatal rat lungs (127). A mechanistically important link to the pathways discussed in Section 8 is the Nrf2-NLRP3 axis: Nrf2 activation suppresses ROS accumulation, which in turn reduces NLRP3 inflammasome assembly, gasdermin D-mediated pyroptosis, and IL-1β/IL-18 release; Nrf2 knockout in BPD mice markedly worsens pyroptosis and impairment of alveolarization (121).

Several pharmacological Nrf2 activators have shown efficacy in neonatal hyperoxia models. TBHQ activated Nrf2/HO-1, reduced ROS, suppressed NLRP3-driven pyroptosis, and restored alveolar structure in hyperoxia-exposed neonatal mice, with the converse phenotype in Nrf2-knockout animals exposed to hyperoxia alone (121). CDDO-imidazolide provided potent Nrf2-dependent protection against hyperoxic acute lung injury in adult mice (attenuating hemorrhage, edema, and inflammatory infiltration) with no protection in Nrf2-knockout counterparts (128). Maternal sulforaphane administration attenuated hyperoxia-induced neutrophilia, edema, oxidation, and alveolarization arrest in newborn mice, with the attenuation more marked in Nrf2-deficient animals, indicating both Nrf2-dependent and Nrf2-independent protection (129). Rapamycin, an autophagy agonist, enhanced Nrf2 nuclear translocation and improved alveolar simplification in neonatal rats (130). Dimethyl fumarate, a fumaric acid ester that activates Nrf2 through Keap1 modification, recovered chord length, septal thickness, and radial alveolar counts in both male and female neonatal mice while reducing cell death and increasing cell proliferation and lung vascularization — the first demonstration that a systemically administered Nrf2 activator can promote resolution of established experimental moderate BPD (131). A notable caveat is that responses are strain- and route-dependent, underscoring the need to evaluate delivery timing, dose, and genetic background before clinical translation (129).

#### 8.2.2 Trained immunity and epigenetic reprogramming

Unlike the antigen-specific memory of the adaptive immune system, trained immunity is a form of innate immune memory: much as prior exposure can prime a cell for a faster or altered response, a monocyte that has survived one inflammatory encounter rewires its epigenome and metabolism to respond more vigorously (or differently) to the next challenge. In BPD, this rewiring occurs in a preterm myeloid compartment already shaped by chorioamnionitis, hyperoxia, and developmental immaturity, with consequences that are only beginning to be understood. Although direct evidence in human BPD remains limited, convergent findings from neonatal models and related inflammatory disorders support trained immunity as a biologically plausible contributor.

Trained immunity refers to the functional reprogramming of innate immune cells (primarily monocytes and macrophages) through epigenetic modifications (H3K4me3 at gene promoters, H3K4me1 and H3K27ac at enhancers) and metabolic shifts (enhanced glycolysis, altered mevalonate pathway) following an initial inflammatory stimulus, resulting in augmented or altered responses to subsequent unrelated challenges (**Figure 14**) (132, 133). This reprogramming occurs both in peripheral myeloid cells and at the level of bone marrow hematopoietic stem and progenitor cells, explaining why the trained phenotype persists beyond the short circulating half-life of monocytes themselves (134).

**Figure 14:**
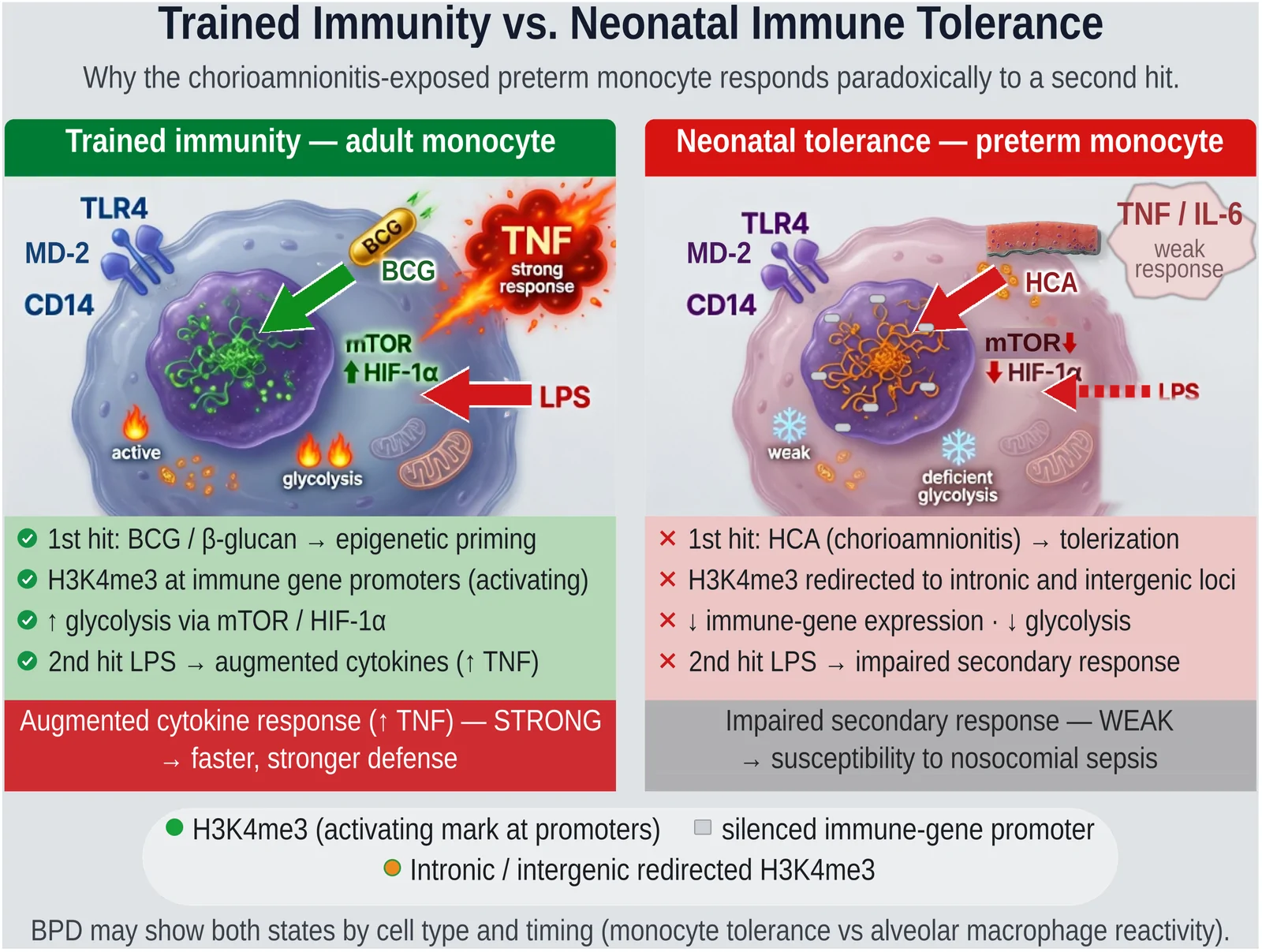
Trained Immunity vs. Neonatal Immune Tolerance in Preterm Monocytes. Side-by-side comparison of the epigenetic and metabolic mechanisms distinguishing trained immunity (left, adult BCG/β-glucan-primed monocyte) from chorioamnionitis-induced neonatal immune tolerance (right, preterm monocyte). In trained immunity, first-exposure epigenetic reprogramming places H3K4me3 at immune gene promoters, enabling an augmented cytokine response to secondary stimulation. In neonatal tolerization, chorioamnionitis redirects H3K4me3 away from immune gene promoters toward intronic and intergenic loci; this paradoxical activation mark is associated with reduced immune gene expression and impaired secondary responses to LPS and nosocomial pathogens. Metabolic correlates are shown for each state. H3K4me3, histone H3 lysine 4 trimethylation; mTOR, mechanistic target of rapamycin; HIF-1α, hypoxia-inducible factor 1-alpha; BCG, Bacillus Calmette–Guérin; TLR, Toll-like receptor; TNF, tumor necrosis factor; LPS, lipopolysaccharide; HCA, histologic chorioamnionitis; MD-2, myeloid differentiation factor 2; CD14, cluster of differentiation 14.

Neonatal innate immune training diverges from the adult paradigm (**Figure 14**). BCG-trained adult monocytes show the expected enhanced TNF production upon LPS restimulation; in contrast, BCG-trained newborn monocytes show LPS tolerance, with significantly diminished production of TNF, IL-10, and CCL5 — accompanied by markedly impaired BCG-induced lactate production, indicating deficient glycolytic reprogramming (135). In a separate *in vitro* comparison, however, neonatal and adult monocytes adapted similarly after both BCG and β-glucan exposure, with β-glucan increasing IL-6, IL-10, and TNF production capacity in both age groups — a result that does not reproduce the neonatal BCG tolerance above and leaves open whether the discrepancy reflects the training stimulus, the readout, or the donor population (136). Preterm macrophages primed by hyperoxia and subsequently stimulated with LPS show an exaggerated pro-inflammatory response compared to LPS stimulation alone, mediated in part by downregulation of the transcriptional regulators *Egr2* (under hyperoxia) and *Gfi1* (under hypoxia) — mirroring trained immunity in its hallmark feature of augmented responsiveness to secondary inflammatory challenge (137).

Chorioamnionitis — present in the great majority of placentas delivered at 21–24 weeks’ gestation (≈94%) and in a substantial minority thereafter and discussed as a first-hit insult in Section 4 — produces global remodeling of the neonatal monocyte histone landscape. ChIP-seq and RNA-seq analyses demonstrate that chorioamnionitis-exposed neonatal monocytes exhibit a net increase in total H3K4me3, primarily at intronic and intergenic rather than canonical promoter sites — but this epigenetic activation paradoxically associates with decreased immune gene expression and a less robust inflammatory response to secondary challenge, a pattern more consistent with immune tolerance than classical trained immunity (26). This tolerized phenotype may explain the well-documented increased susceptibility to postnatal sepsis in chorioamnionitis-exposed neonates. Complementary SMaRT (Signatures of Macrophage Reactivity and Tolerance) analysis confirms that cord blood monocytes from infants who develop BPD exhibit a tolerant phenotype at birth that persists in peripheral blood monocytes throughout the neonatal period, while lung macrophages show early reactivity in infants who develop BPD (days 1–7), and macrophages from infants with placental inflammatory disease converge after day 14 toward the phenotype associated with their placental disease; tracheal aspirate data further show that infants with histological chorioamnionitis have lower M1 and higher M2 macrophage levels, consistent with myeloid tolerization (45, 138).

*Ureaplasma* exposure may additionally prime the neonatal myeloid compartment through TLR2/TLR6 and TLR9 activation — pathways capable of inducing trained immunity in other inflammatory contexts (139). *Ureaplasma* isolates stimulate pro-inflammatory CC chemokines and MMP-9 in neonatal monocytes, and co-stimulation of LPS-primed neonatal monocytes with *Ureaplasma parvum* enhances LPS-induced MCP-1 and MMP-9 expression — demonstrating that *Ureaplasma* can directly modulate monocyte responsiveness to subsequent stimuli (140). In a rhesus macaque model, intra-amniotic *Ureaplasma parvum* decreased fetal Tregs and polarized them toward a Th1 phenotype, indicating that myeloid reprogramming by *Ureaplasma* begins *in utero* (33, 141). The emerging picture across these studies is one of a paradoxical epigenetic state: global H3K4me3 activation marks are increased in chorioamnionitis-exposed neonatal monocytes, yet functional immune gene transcription is suppressed — creating a myeloid compartment simultaneously tolerized to some stimuli and hyperresponsive to others depending on the nature and timing of the secondary challenge (26). This epigenetic heterogeneity likely contributes to the variable inflammatory phenotypes observed across BPD endotypes and may ultimately inform biomarker strategies for identifying infants most likely to benefit from targeted immunomodulatory therapy.

## 9 Discussion

### 9.1 From mechanism to target

A mechanistic implication of the account assembled above is that an agent acting broadly on inflammation is unlikely to be matched to the dominant inflammatory pathway or developmental stage present in any individual infant (**Figure 15**). This is an argument about mechanism, not a verdict on efficacy, and current interventions are not one therapeutic category: they differ in mechanism, indication and evidence base — surfactant addresses a biophysical deficit rather than inflammation, caffeine is a respiratory stimulant with the additional anti-inflammatory actions noted in Section 5.1.1, corticosteroids act broadly on inflammatory transcription — and this review evaluates the efficacy of none of them. The account assembled above is convergent by construction, and convergence yields a finite list of nodes at which the cascade might be interrupted. The nodes are appraised below across four dimensions rather than ranked, because they occupy different biological levels — a receptor, an inflammasome, a cytokine, a lipid-mediator system, a transcription factor, a cellular state, the microbiome — across which no single ordinal scale is defensible. The dimensions are: whether the pathway has been shown to be necessary (not merely present) in a neonatal lung injury model with alveolarization endpoints; whether it has been demonstrated in preterm human airway or blood; and whether its perturbation protects vascular as well as alveolar development. Each node is also assigned the developmental phase in which it is engageable, since a target that is mechanistically sound but only reachable after the injury it causes is of limited value. The phases used here are those established in the preceding sections: antenatal priming (chorioamnionitis and FIRS, before delivery), acute initiation (approximately the first postnatal week, when TLR-driven signaling is maximal), amplification and failed resolution (roughly days 7 to 28), and remodeling (beyond day 28, when TGF-β-driven fibrosis and vascular remodeling predominate). The appraisal reflects mechanistic and model evidence, not clinical efficacy, and the nodes are presented in the order in which they arise in the cascade rather than in order of merit. TLR2/TLR4 is presented first as the shared entry point of the multiple-hit model, not as the best-validated effector node — that distinction belongs to the NLRP3/IL-1 axis, where demonstrated genetic necessity in an alveolarization model is strongest (**Table 2**).

**Figure 15:**
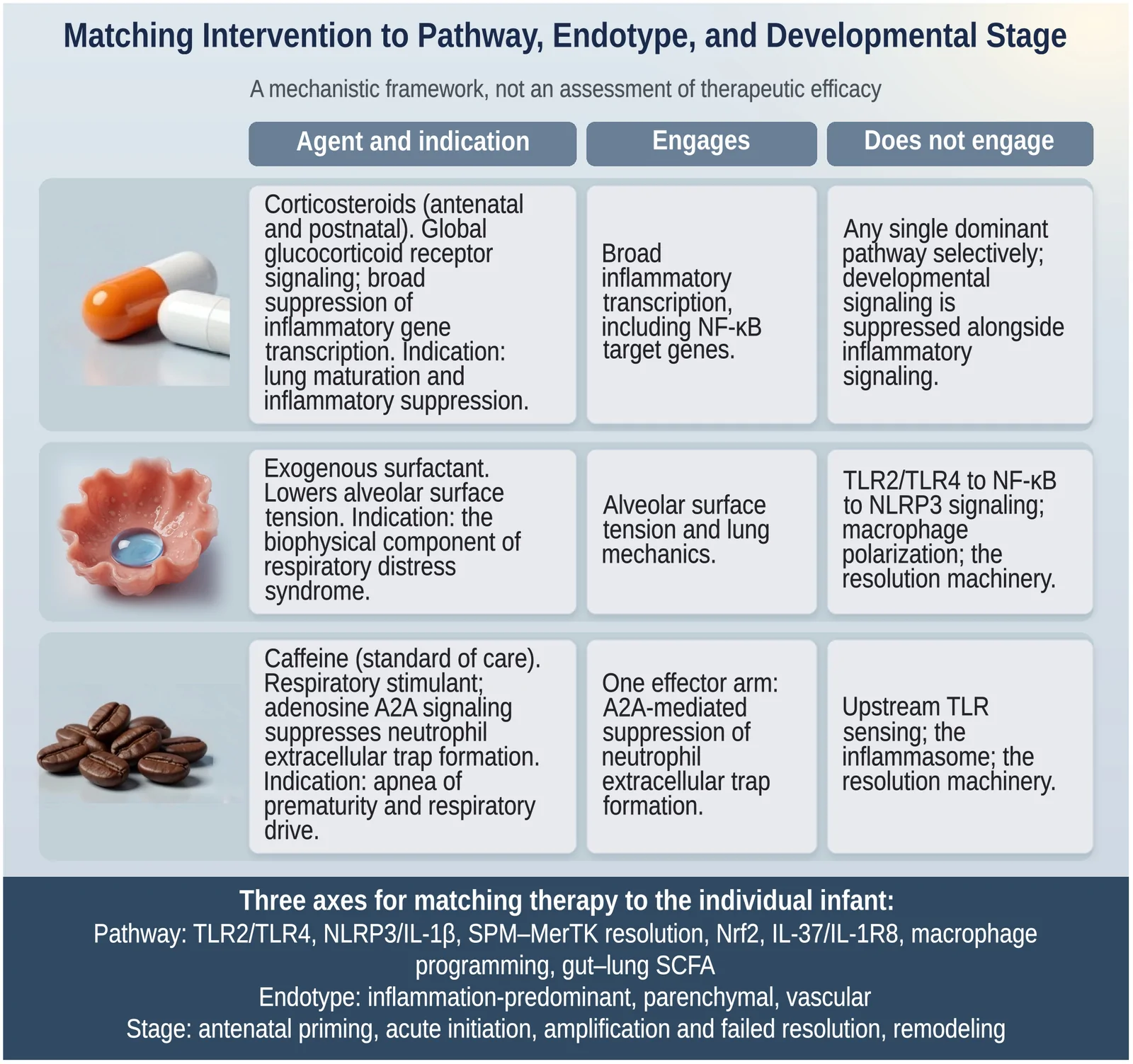
The rationale for pathway-, endotype- and stage-matched intervention. Which pathway dominates, which endotype is present, and which developmental stage the lung has reached differ between infants, so an agent acting broadly on inflammation is unlikely to be matched to any individual infant’s biology. For three interventions in current use the figure sets out, descriptively, which parts of the cascade described in this review each does and does not engage. This is not an assessment of any of them: they differ in mechanism, indication and evidence base, they are not a single therapeutic category, and therapeutic efficacy is not evaluated anywhere in this review. The figure summarizes only the mechanistic rationale for a precision-matched alternative. BPD, bronchopulmonary dysplasia; A2A, adenosine A2A receptor; IL, interleukin; NETs, neutrophil extracellular traps; NF-κB, nuclear factor kappa B; NLRP3, NOD-, LRR- and pyrin domain-containing protein 3; TLR, Toll-like receptor; SPM, specialized pro-resolving mediator; MerTK, Mer receptor tyrosine kinase; Nrf2, nuclear factor erythroid 2-related factor 2; IL-1R8, interleukin-1 receptor 8 (SIGIRR/TIR8); SCFA, short-chain fatty acid.

1. TLR4 and TLR2 sensing (strongest mechanistic evidence; engageable in antenatal priming and acute initiation). The receptors are the shared entry point of the entire multiple-hit model: microbial ligands, hyperoxia-generated DAMPs, hyaluronan fragments, and HMGB1 all signal through them; TLR pathway polymorphisms modify human BPD risk; and TLR2 mediates the *Ureaplasma*-driven arm of the disease. TLR4-null neonatal mice are protected from impaired alveolarization, though genetic-necessity evidence in a neonatal alveolarization model remains broader for the downstream NLRP3/IL-1 axis than for the receptors themselves; in adult oxidant lung injury TLR4 signaling is reported to be protective through a TRIF-dependent, MyD88-independent Hsp70 arm (5–7, 142). The constraint is a well-described paradox: the same receptors carry the epithelial survival and repair signals, so the target is the injurious output rather than the receptor itself. A further constraint is that broad TLR blockade would be expected to increase infection risk in an already immunocompromised preterm host, reinforcing that the aim is selective modulation of injurious signaling rather than receptor silencing.
2. The NLRP3 inflammasome (strongest mechanistic evidence; engageable from acute initiation through amplification). NLRP3 is necessary, not merely associated: NLRP3-null neonatal mice are protected from hyperoxia-induced impairment of alveolarization, CD44 is required for TLR4-mediated NLRP3 activation in BPD models, and caspase-1 inhibition improves alveolar and vascular development while simultaneously protecting the developing brain (4, 5, 143). It is the point at which sensing is converted into an effector cytokine, and it is the node at which the largest number of endogenous brakes (IL-37, Nrf2, resolvin D2, SCFAs) converge.
3. IL-1β and IL-1 receptor signaling (strong mechanistic and preclinical evidence; engageable from acute initiation through amplification). IL-1 receptor antagonism prevents BPD-like structural disintegration in newborn mice exposed to perinatal inflammation plus hyperoxia at moderate but not severe hyperoxia and, in a parallel experiment, prevents the pulmonary vascular disease that accompanies it, with sustained benefit to day 60 (144, 145). IL-1β is detectable in human lung lavage from day 1 and is the effector through which NLRP3 acts, which makes it the most directly druggable point of the axis and the one with the clearest dual (alveolar and vascular) protective signature. Any clinical use of IL-1 blockade in this population would need to weigh the infectious-risk and developmental implications of suppressing IL-1 signaling in a preterm host, even though therapeutic efficacy and safety are formally out of scope here.
4. The resolution machinery: SPM biosynthesis, FPR2/ALX signaling, and MerTK-dependent efferocytosis (strong mechanistic rationale, no human validation; engageable during amplification and failed resolution). This is the node that follows most directly from the central thesis of this review, and it is also the least characterized in human disease. The four-point failure of the SPM program, and receptor-specific rescue of it, are documented in adult models of failed resolution (93, 146); the preterm-lung evidence remains indirect (9). What is missing is any measurement of the mediators themselves in preterm infants, which places this node one descriptive study away from a much stronger evidentiary position.
5. The IL-37/IL-1R8 axis (strong mechanistic rationale, no neonatal lung data; engageable across initiation and amplification). IL-37 is the principal broadly anti-inflammatory member of the IL-1 family and, in mouse models, suppresses NLRP3 at both the priming and the activation step, extracellularly through the IL-18Rα/IL-1R8 receptor pair (97, 100). Its convergence with the central pathological node of BPD is exact. It has never been measured in a preterm infant, however, and no hyperoxia-based neonatal model with alveolarization endpoints has been reported.
6. Nrf2–Keap1–ARE antioxidant defense (good mechanistic evidence; engageable in acute initiation, and plausibly antenatally). The preterm lung enters a hyperoxic environment with an immature Nrf2 response and low HO-1, NQO1, and GCLC reserve; Nrf2 deficiency worsens NLRP3-mediated pyroptosis and impairs alveolar growth, and pharmacological Nrf2 activation limits hyperoxic injury in neonatal mouse models (121, 125). Nrf2 is best understood as an upstream brake on signal 2 of the inflammasome rather than as an independent axis.
7. Macrophage polarization and trained immunity (the integrating node; engageable across all phases, and the only node that speaks to durability). M1 persistence is the cellular phenotype through which every upstream pathway above expresses itself, and epigenetic myeloid reprogramming is the proposed mechanism by which a neonatal insult persists beyond the neonatal period (26, 132–134). Human lung data complicate this: in the LungMAP series, macrophages move from reactive in evolving BPD to tolerant in established and healed BPD, while dendritic cells remain reactive at 15–21 months (138). Polarization is a state rather than a receptor, which makes it an excellent pharmacodynamic readout and a difficult primary target; the durability problem it poses is, however, the strongest argument for intervening early rather than late.
8. The gut-lung axis and SCFA signaling (moderate mechanistic evidence; engageable from the first postnatal days and uniquely modifiable without a drug). SCFAs restrain NF-κB and NLRP3 through HDAC inhibition and GPR43 signaling, and the preterm SCFA deficit is imposed by early antibiotic exposure rather than by prematurity alone, though this restraint is context-dependent and can reverse under active TLR co-stimulation (147–149). This is the only node on this list that can be moved by changes in ordinary neonatal practice, which gives it a translational value out of proportion to the strength of its preclinical evidence.

Two structural features of this list deserve emphasis. First, the nodes are not independent: TLR4 primes NLRP3, NLRP3 produces IL-1β, and IL-37, Nrf2, SPMs, and SCFAs all converge on NLRP3 as brakes. An agent acting at any one of them will move the others, and the failure to account for this is one reason single-target trials in BPD have been difficult to interpret. Second, the phase in which a node is engageable is a property of the biology and not of the drug: TLR-directed strategies must act before the cascade they modulate is established, whereas resolution-directed strategies are by definition late, and no agent can restore septa that have already failed to form. One implication follows directly from the failed-resolution frame: trials built on these nodes should incorporate endpoints that capture resolution biology and alveolar-vascular growth — resolution-mediator and efferocytosis readouts, and structural or imaging measures of alveolarization and vascular development — rather than relying on the oxygen requirement at 36 weeks alone, with the timing of therapy (early initiation-blockers versus later resolution agonists) matched to the phase each node occupies.

### 9.2 Future research priorities

Several questions raised here are directly answerable. First, can SPM deficiency, efferocytosis capacity, or IL-37/Nrf2 activity be measured prospectively in preterm blood or airway samples rather than inferred retrospectively from outcome? Second, can BPD endotypes be identified early enough to guide therapy, rather than reconstructed after the fact from data collected for other purposes? Third, for how long is early immune programming (trained immunity, epigenetic reprogramming of the fetal myeloid compartment, FIRS-associated set-point shifts) reversible, and does that window close before or after BPD becomes clinically apparent? Fourth, which of the biomarkers cataloged here are predictive of therapeutic response rather than merely diagnostic or prognostic, since only predictive biomarkers can allocate therapy at the bedside? Several methodological priorities follow: gestational-age-adjusted endotyping of funisitis and FIRS with multi-omic myeloid profiling, isolating the fetal-response contribution from GA confounding; uniform application of the 2019 Jensen BPD grades across biomarker studies, so effect estimates are comparable; standardized airway lipidomics for SPMs and their PUFA precursors, which remain unharmonized; a direct test of the central thesis — whether failure of resolution is necessary, sufficient, or merely synergistic with the initiating hit; and sex-stratified immune trajectories. Answering these questions would do more to enable precision immune-developmental therapy than any single new mechanistic discovery.

### 9.3 Limitations of this review

Several limitations warrant explicit acknowledgment. The first concerns methodology. As a narrative review, the synthesis is qualitative, with no quantitative pooling of effect estimates. A further caveat is intrinsic to the convergent frame: convergence is asserted at the shared MyD88–NF-κB–NLRP3 node, not at the receptor, and the sensing layer remains heterogeneous. The antiviral TLR3/TLR7/RIG-I–IRF3/7 arm runs parallel to that node, and one receptor can carry opposing outputs segregated by adaptor and stage — TLR4 deletion protects neonatal mice from impaired alveolarization, whereas in adult hyperoxia a TLR4 arm signaling through TRIF and independently of MyD88 is cytoprotective (142). Whether the shared node is the dominant route in most infants, or the best-studied one, is untested in preterm humans.

The second limitation concerns the strength and provenance of the underlying evidence. Much of the mechanistic account assembled here rests on preclinical data: neonatal rodent hyperoxia models, two-hit lipopolysaccharide-plus-hyperoxia models, and preterm lamb and baboon models, in which the injury is applied in a standardized fashion that human prematurity is not. Differences in species, developmental timing, and injury paradigm complicate cross-study comparison, and the well-recognized gap between preclinical mechanism and clinical relevance counsels caution. Several of the pathways given prominence here, including the SPM system, the IL-37/IL-1R8 axis, and the gut-lung SCFA axis, have never been measured directly in preterm human airway or blood, and the human evidence for them is inferential. Where human data do exist, they are largely associative, consisting of biomarker–outcome correlations rather than demonstrations of causal necessity. The endotype and biomarker tools described are research-grade and are not validated for clinical or bedside use. The failed-resolution framework should therefore be read as a hypothesis resting largely on E2–E4 evidence, not as an established mechanism in human BPD.

The third limitation concerns scope and timeliness. This review is limited to English-language sources, which may introduce individual and linguistic bias. Racial and ethnic disparities in BPD incidence and severity are large and well documented, but their immunological basis has not been studied in a way that would support mechanistic synthesis, and they are therefore not treated as a separate section here; this is an acknowledged omission rather than a judgment that the question is unimportant. Finally, the immunology of BPD is advancing rapidly, and the analysis reflects evidence available up to a 2026 knowledge cutoff; subsequent findings may refine or supersede the interpretations offered here.

## 10 Conclusion

BPD is, at its mechanistic core, an immune-developmental disease. The preterm lung is delivered into an inflammatory environment at the late canalicular to early saccular transition, before alveolarization and microvascular growth are complete, and the heterogeneous insults it then encounters (chorioamnionitis and FIRS, *Ureaplasma*, hyperoxia, mechanical ventilation, sepsis, and intestinal dysbiosis) are read through a small number of shared innate sensors. TLR2 and TLR4 signal through MyD88 and NF-κB to prime and activate the NLRP3 inflammasome; IL-1β and IL-18 are released; M1-polarized alveolar macrophages persist; and the growth factor program on which septation and angiogenesis depend is suppressed. The pathology of BPD is the pathology of a developmental program interrupted by a signaling cascade that shares its tissue and part of its vocabulary.

What converts injury into impaired development, however, is not the initiating hit but the failure of the response to terminate. Resolution in the preterm lung is vulnerable at multiple levels, and different infants may fail at different points: the substrate for SPMs is depleted, 5-lipoxygenase is mislocalized toward leukotriene rather than lipoxin synthesis, efferocytosis fails and thereby deepens its own failure, and the endogenous brakes that should restrain the inflammasome (IL-37, Nrf2, resolvin D2, complement regulators, and microbially derived SCFAs) are immature, deficient, or iatrogenically removed. Inflammation in BPD is therefore not merely excessive; it is unterminated. This distinction is not semantic, because a disease of excess invites suppression while a disease of failed resolution invites restoration, and the two imply different targets, different timing, and different endpoints.

The disease is also not uniform, and the review has been explicit about in whom and for how long it operates. Male sex, hemizygosity at X-linked immune loci, sex-specific alveolar macrophage metabolism, TLR pathway polymorphisms, deficient transplacental IgG transfer, disrupted microbiome seeding, and endotype all modify the translation of a given insult into disease, and the inflammation-predominant, parenchymal, and vascular endotypes differ in the pathways that drive them — a conceptual partition, not yet a validated clinical classification. The developmental disruption, moreover, does not end at 36 weeks postmenstrual age. Trained myeloid programming, persistent Th17 skew, a mechanistic basis for concern about impaired vaccine responses, and inflammasome-mediated lung-brain crosstalk carry the immune signature of BPD into childhood and adult life, which means that the outcome by which BPD is conventionally judged measures a snapshot of a process that continues for decades.

The mechanistic account therefore yields a specific and finite set of targets: the TLR2/TLR4 sensing layer, the NLRP3 inflammasome and its IL-1β output, the resolution machinery, the IL-37/IL-1R8 axis, Nrf2, macrophage polarization and its epigenetic durability, and the gut-lung SCFA axis, each engageable in a defined developmental phase. Three of these have never been measured in the preterm human airway or blood, which is the principal evidence gap identified in this review and the one most readily addressable with existing sampling and assay methods. Whether any of these targets can be engaged to clinical benefit, in what phase, and in which endotype, is a question this review does not attempt to settle. Taken together, these findings argue for a broader reframing of BPD research and care. The field has largely asked how to suppress inflammation in the preterm lung; the evidence assembled here suggests the more productive question is how to restore the developmental program of resolution that inflammation was always meant to yield to. Under this view, BPD is better understood not as a disease to be treated by immune suppression, but as a developmental disorder of immune timing to be addressed by restoring the resolution program — matched to the pathway, endotype, and developmental window in which each infant’s disease is unfolding. Because BPD is multifactorial, such immune-directed strategies will need to be integrated with — not substituted for — optimal ventilation, oxygen targeting, and nutrition rather than pursued in isolation. That shift, from generic anti-inflammation to precision immune-developmental medicine, is the paradigm this review argues the field is now positioned to make.

## Glossary

A2A: adenosine A2A receptor
ACS: antenatal corticosteroid
AEC/AEC II: alveolar epithelial cell/alveolar epithelial cell, type II
AKT: protein kinase B (AKT serine/threonine kinase)
ALX/FPR2: lipoxin A4 receptor/formyl peptide receptor 2
aOR: adjusted odds ratio
AQP5: aquaporin 5
ARDS: acute respiratory distress syndrome
ARE: antioxidant response element
AREG: amphiregulin
Arg-1: arginase 1
ASC: apoptosis-associated speck-like protein containing a CARD
ATP: adenosine triphosphate
AVR-48: chitin-derived small-molecule TLR4 modulator (investigational, AyuVis Research)
BAL/BALF: bronchoalveolar lavage/bronchoalveolar lavage fluid
Batf3: basic leucine zipper ATF-like transcription factor 3
BCG: Bacillus Calmette–Guérin
BPD: bronchopulmonary dysplasia
BPD36: bronchopulmonary dysplasia at 36 weeks postmenstrual age
BPD-PH: BPD-associated pulmonary hypertension
β-catenin: beta-catenin (Wnt pathway effector)
CA: chorioamnionitis
CARD: caspase activation and recruitment domain
CCL/CCR: C-C motif chemokine ligand/receptor (e.g., CCL2, CCL5, CCL13, CCL24
CD: cluster of differentiation (e.g., CD4, CD11b, CD11c, CD14, CD16, CD44, CD56, CD74, CD86, CD103, CD206)
cDC1/pDC: conventional type 1 dendritic cell/plasmacytoid dendritic cell
CDDO: 2-cyano-3,12-dioxooleana-1,9(11)-dien-28-oic acid (synthetic triterpenoid; e.g., CDDO-imidazolide)
cGAS/STING: cyclic GMP-AMP synthase/stimulator of interferon genes (cytosolic DNA-sensing pathway)
ChIP-seq: chromatin immunoprecipitation sequencing
CI: confidence interval
Clec9a: C-type lectin domain family 9 member A (DNGR-1)
c-MYC: MYC proto-oncogene protein
CO: carbon monoxide
COL1A1/COL1A2/COL3A1: collagen type I α1/type I α2/type III α1 (genes)
COX-2: cyclooxygenase-2
CRP: C-reactive protein
CT: computed tomography
CXCL2/CXCR2: C-X-C motif chemokine ligand 2/receptor 2
DAMP: damage-associated molecular pattern
DC: dendritic cell
DHA/EPA: docosahexaenoic acid/eicosapentaenoic acid
DNA: deoxyribonucleic acid
DSS: dextran sulfate sodium
ECM: extracellular matrix
EGFR: epidermal growth factor receptor
Egr2/Gfi1: early growth response 2/growth factor independent 1
ERK: extracellular signal-regulated kinase
FcγRIII: Fcγ
FGF: fibroblast growth factor
FiO₂: fraction of inspired oxygen
FIR/MIR: fetal/maternal inflammatory response
FIRS: fetal inflammatory response syndrome
FLT3L: FMS-like tyrosine kinase 3 ligand (also written Flt3L)
FOXP3: forkhead box P3 (regulatory T-cell transcription factor)
G0F: agalactosylated, core-fucosylated IgG Fc glycoform
GA: gestational age
GCLC: glutamate-cysteine ligase catalytic subunit
GM-CSF: granulocyte-macrophage colony-stimulating factor
GPR18/GPR32: G-protein-coupled receptors 18/32 (resolvin receptors)
GPR43/GPR109a: G-protein-coupled receptors 43/109a (SCFA receptors)
GRADE: Grading of Recommendations Assessment, Development and Evaluation
GSDMD: gasdermin D
GSDMD-N: gasdermin D N-terminal (pore-forming) fragment
GSH: glutathione (reduced)
H3K27ac: histone H3 lysine 27 acetylation
H3K4me1: histone H3 lysine 4 monomethylation
H3K4me3: histone H3 lysine 4 trimethylation
HCA: histologic chorioamnionitis
HDAC: histone deacetylase
HETE: hydroxyeicosatetraenoic acid (e.g., 15S-HETE)
HIF-1α: hypoxia-inducible factor 1-alpha
HLA-DR: human leukocyte antigen DR
HMGB1: high mobility group box 1
HO-1: heme oxygenase-1
ICAM-1: intercellular adhesion molecule 1
IFN: interferon
IgG: immunoglobulin G
IL: interleukin (e.g., IL-1β, IL-6, IL-10, IL-17A, IL-37)
IL-1R8: interleukin-1 receptor 8 (SIGIRR/TIR8)
IL-1Ra: interleukin-1 receptor antagonist (anakinra)
ILC2: type 2 innate lymphoid cell
iNOS: inducible nitric oxide synthase
IRAK: interleukin-1 receptor-associated kinase
IRF3/IRF4: interferon regulatory factor 3/4
JNK: c-Jun N-terminal kinase
KATP/SUR1: ATP-sensitive potassium channel/sulfonylurea receptor 1
Keap1: Kelch-like ECH-associated protein 1
LGR6: leucine-rich repeat-containing G-protein-coupled receptor 6
LOX: lipoxygenase
LPS: lipopolysaccharide
LTA4: leukotriene A4
LTB4/LXA4: leukotriene B4/lipoxin A4
M1/M2: classically/alternatively activated macrophage
MAPK: mitogen-activated protein kinase
MBA: multiple-banded antigen (Ureaplasma)
MBL: mannose-binding lectin/its gene
MCP-1: monocyte chemoattractant protein-1 (CCL2)
MerTK: Mer receptor tyrosine kinase
MICA/MICB: MHC class I polypeptide-related sequence A/B
MIF: macrophage migration inhibitory factor
MIP-2: macrophage inflammatory protein 2 (CXCL2)
miR/miRNA: microRNA
MMP: matrix metalloproteinase
Mon1/Mon3: classical/non-classical monocyte subsets
mRNA: messenger RNA
mTOR/mTORC1: mechanistic target of rapamycin/complex 1
MV: mechanical ventilation
MyD88: myeloid differentiation primary response 88
NEC: necrotizing enterocolitis
NET: neutrophil extracellular trap
NF-κB: nuclear factor kappa B
NICHD: Eunice Kennedy Shriver National Institute of Child Health and Human Development
NK: natural killer (cell)
NKG2D: natural killer group 2 member D (activating receptor)
NLRP1: NOD-, LRR-, and pyrin domain-containing protein 1
NLRP3: NOD-, LRR-, and pyrin domain-containing protein 3 (inflammasome)
NQO1: NAD(P)H quinone dehydrogenase 1
Nrf2: nuclear factor erythroid 2-related factor 2
OR: odds ratio
OX40L: OX40 ligand
P2X7: P2X purinoceptor 7 (ATP-gated cation channel)
PAMP: pathogen-associated molecular pattern
PDA: patent ductus arteriosus
PH: pulmonary hypertension
PI3K: phosphoinositide 3-kinase
PLA2: phospholipase A2
PMA: postmenstrual age
PMN: polymorphonuclear neutrophil
PND: postnatal day
PTEN: phosphatase and tensin homolog
PU.1: purine-rich box 1 (SPI1) transcription factor
PUFA: polyunsaturated fatty acid
RIG-I: retinoic acid-inducible gene I (cytosolic RNA sensor)
RNA-seq/siRNA: RNA sequencing/small interfering RNA
RORγt/RORC: RAR-related orphan receptor gamma t/its gene
ROS: reactive oxygen species
RR: risk ratio
RSV: respiratory syncytial virus
RvD1: resolvin D1
RvD2: resolvin D2
RvE1: resolvin E1
S100A8: S100 calcium-binding protein A8
SCFA: short-chain fatty acid
sICAM-1: soluble intercellular adhesion molecule 1
SIGIRR: single immunoglobulin IL-1-related receptor (TIR8)
SIRT1: sirtuin 1 (NAD-dependent deacetylase)
Smad3/p-Smad3: SMAD family member 3/phosphorylated Smad3
SMaRT: Signatures of Macrophage Reactivity and Tolerance
SNP: single nucleotide polymorphism
SPC: surfactant protein C
SPM: specialized pro-resolving mediator
ST2/sST2: interleukin-1 receptor-like 1 (IL-1RL1)/its soluble form
STAT1/STAT3: signal transducer and activator of transcription 1/3
TBHQ: tert-butylhydroquinone (Nrf2 activator)
TGF-β: transforming growth factor beta
Th1/Th2: T helper 1 cell/T helper 2 cell
Th17/Treg: T helper 17 cell/regulatory T cell
TIR: Toll/interleukin-1 receptor (domain)
TLR: Toll-like receptor (e.g., TLR2, TLR4)
TNF-α: tumor necrosis factor alpha
TP53: tumor protein p53
TRIF: TIR-domain-containing adaptor protein inducing interferon-β
VEGF/VEGFR2: vascular endothelial growth factor/its receptor 2
Wnt: wingless-related integration site (signaling pathway)
WNT3a: Wnt family member 3A.
